# Prevalence, comorbidity, and breed differences in canine anxiety in 13,700 Finnish pet dogs

**DOI:** 10.1038/s41598-020-59837-z

**Published:** 2020-03-05

**Authors:** Milla Salonen, Sini Sulkama, Salla Mikkola, Jenni Puurunen, Emma Hakanen, Katriina Tiira, César Araujo, Hannes Lohi

**Affiliations:** 1 Department of Medical and Clinical Genetics, University of Helsinki, Helsinki, Finland; 2 Department of Veterinary Biosciences, University of Helsinki, Helsinki, Finland; 3 Folkhälsan Research Center, Helsinki, Finland

**Keywords:** Behavioural ecology, Animal behaviour, Epidemiology, Comorbidities

## Abstract

Behaviour problems and anxieties in dogs decrease their quality of life and may lead to relinquishment or euthanasia. Considering the large number of pet dogs and the commonness of these problematic behaviours, a better understanding of the epidemiology and related molecular and environmental factors is needed. We have here studied the prevalence, comorbidity, and breed specificity of seven canine anxiety-like traits: noise sensitivity, fearfulness, fear of surfaces and heights, inattention/impulsivity, compulsion, separation related behaviour and aggression with an online behaviour questionnaire answered by dog owners. Our results show that noise sensitivity is the most common anxiety-related trait with a prevalence of 32% in 13,700 Finnish pet dogs. Due to the high prevalence of noise sensitivity and fear, they were the most common comorbidities. However, when comparing the relative risk, the largest risk ratios were seen between hyperactivity/inattention, separation related behaviour and compulsion, and between fear and aggression. Furthermore, dog breeds showed large differences in prevalence of all anxiety-related traits, suggesting a strong genetic contribution. As a result, selective breeding focusing on behaviour may reduce the prevalence of canine anxieties. Anxious animals may suffer from chronic stress and thus, modified breeding policies could improve the welfare of our companion dogs.

## Introduction

Problematic behaviours can be a threat to dog welfare. Anxious dogs may be more vulnerable to diseases and show decreased lifespan^1^. Satisfaction with the dog’s behaviour may increase attachment to the dog^2^ and problematic behaviours, especially aggressiveness, destructiveness, fearfulness and hyperactivity are a common reason for relinquishment to shelters^3,4^. Problematic behaviours can even lead to euthanasia at a young age^5,6^. Behaviour problems, especially aggressiveness, may be public health concerns^7^. Some of these behaviour problems have been suggested to be analogous, or possibly even homologous to human anxiety disorders^8^, and the study of these spontaneous behaviour problems arising in a shared environment with people may reveal important biological factors underlying many psychiatric conditions. For example, canine compulsive disorder resembles human OCD on both phenotypic and neurochemical level^8^.

Behaviour problems are common in our companion canines^9^. The most common reported behaviour problems include excessive barking, inappropriate elimination, destructiveness, aggression and fearfulness^6,10–13^. The prevalence of noise sensitivity in previous studies has varied between 20% and 50%^14–18^. Around 20–25% of dogs show fearfulness of strangers, dogs or situations^15,16^ and separation anxiety occurs in 14–20% of dogs^15,16,19^. Furthermore, comorbidity between noise sensitivity and separation anxiety has also been observed^15,20^.

Behaviour has a major genetic component^21–27^ and many traits are both phenotypically and genetically correlated^22^. For example, relatives of compulsive dogs are often also affected^28^. Some genomic areas and loci are associated with problematic behaviour, including compulsion^29^, fear and noise sensitivity^30^. Furthermore, problematic behaviour may be influenced by many environmental factors, including, for example, maternal care, owner experience, training and exercise^31–33^. Behaviours are complex traits affected by several genes with small effects, multiple environmental factors varying in effect, and intricate interactions between them^34^.

Many epidemiological cross-sectional studies of behaviour are hindered by a small sample size and breed coverage or limited number of traits for a comprehensive overview. For example, our earlier anxiety survey included only three traits and reached ~3,300 participants^16^. Therefore, we developed here a more comprehensive owner-completed online questionnaire to collect a large data set of companion dogs in the study of canine anxiety. We expanded the study from three to seven anxiety-related traits and acquired four times as many records to assess the prevalence of different anxiety-like behaviour problems in a large home living population of companion canines, comorbidity between the anxiety-related traits, and trait-specific behavioural variation between breeds, which could indicate genetic inheritance.

## Results

### Demography

We examined the epidemiology of seven anxiety-like traits in dogs: noise sensitivity, fear, fear of surfaces, inattention/impulsivity, compulsive behaviour, aggression and separation related behaviour with a comprehensive owner-answered online questionnaire. We collected 13,715 responses in 264 dog breeds. In total, 51.5% of the dogs were female and the age of the dogs varied between 10 weeks and 17 years 10 months (mean 4.7 years). We received more than 200 responses from mixed breed dogs and from 14 breeds: Bernese Mountain Dog, Border Collie, Finnish Lapponian Dog, German Shepherd Dog, Labrador Retriever, Lagotto Romagnolo, Lapponian Herder, Miniature Schnauzer, Rough Collie, Shetland Sheepdog, Smooth Collie, Soft-Coated Wheaten Terrier (labelled Wheaten Terrier), Spanish Water Dog and Staffordshire Bull Terrier (labelled Staff. Bull Terrier). These breeds and mixed breed dogs made up 35% of all dogs in the data. The sex ratio did not significantly differ between these breeds (χ^2^ = 4.17, DF = 14, P = 0.994), but the mean age differed (Kruskal-Wallis χ^2^ = 77.53, DF = 14, P < 0.001): Bernese Mountain Dogs ($\bar{{\rm{x}}}$  = 3.8 years, SD = 2.7) were, on average, younger and Miniature Schnauzers ($\bar{{\rm{x}}}$ = 5.3 years, SD = 3.4) and Wheaten Terriers ($\bar{{\rm{x}}}$ = 5.7 years, SD = 3.7) older than other breeds.

### Prevalence

**Figure 1:**
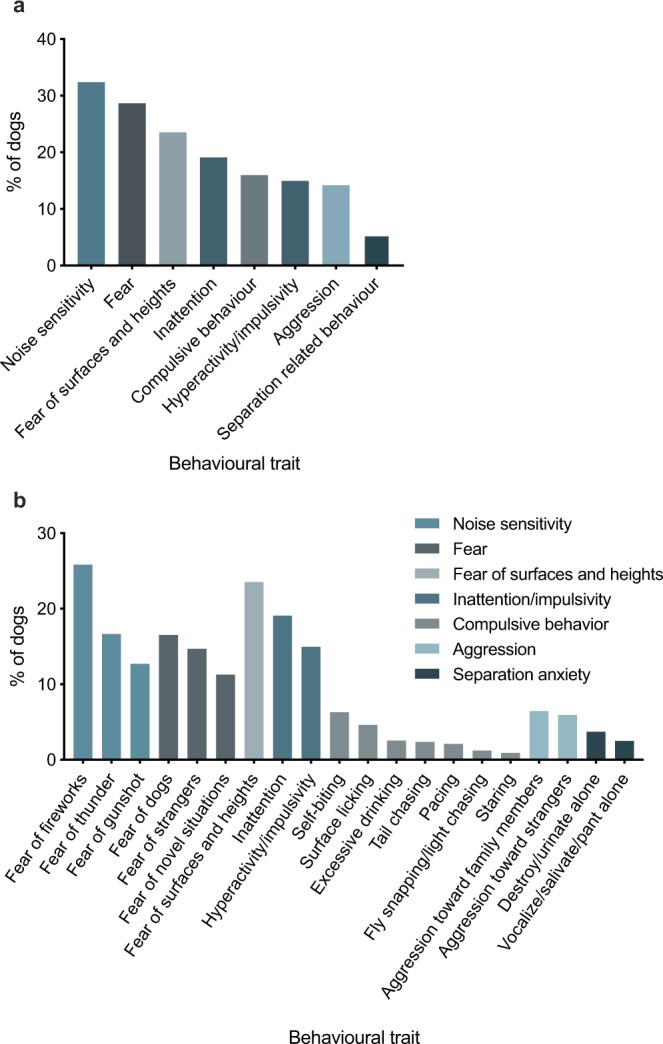
Prevalence of the traits (**a**) and subtraits (**b**) in a sample of 13715 dogs in 264 breeds.

In total, 72.5% of dogs had some kind of highly problematic behaviour. Noise sensitivity was the most common anxiety trait with 32% of dogs being highly fearful of at least one noise (Fig. 1a, Supplementary Table S1). Fear was the second most common trait with a prevalence of 29%. Separation related behaviour and aggression were the most uncommon traits with prevalences of 5% and 14%, respectively.

The prevalence of the subtraits varied (Fig. 1b, Supplementary Table S1). In noise sensitivity, fear of fireworks was the most common subtrait with a prevalence of 26%. When comparing subtraits of fear, fear of other dogs was the most common. Aggression toward human family members was slightly more common than aggression toward strangers. From compulsion subtraits, self-biting was the most commonly reported.

### Comorbidity

**Figure 2:**
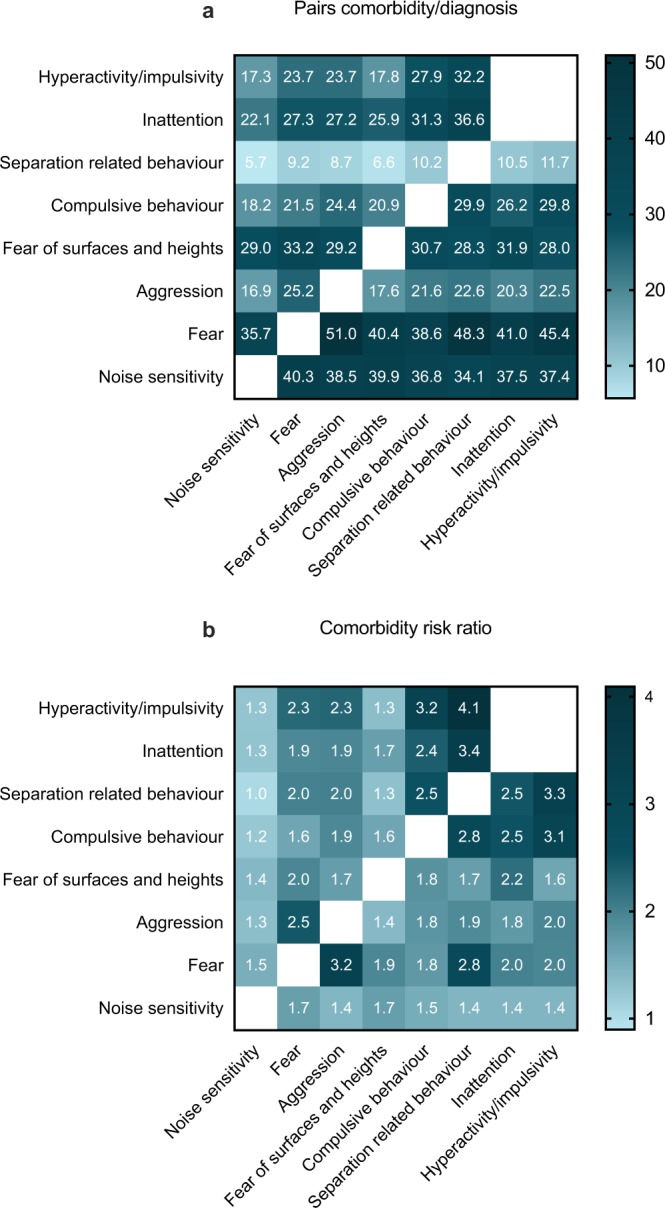
Comorbidity heat maps. Proportion of dogs with pairs of diagnoses using the disorder listed in the column header as the denominator (**a**) and relative risk (**b**). (**a**) Adapted from Goldstein-Piekarski *et al*.^72^.

Many dogs exhibited comorbidities between different anxiety-related traits (Fig. 2a). Most common comorbidity was fear, especially in aggressive and hyperactive/impulsive dogs, and the second most common was noise sensitivity, especially in fearful dogs. However, a somewhat different pattern emerged when comparing the relative risks (Fig. 2b, Supplementary Table S2). Dogs displaying separation related behaviour were 4.1 times more often hyperactive/impulsive and 3.4 times more often inattentive than dogs not displaying separation related behaviour. Similar comorbidities were seen between compulsion and hyperactivity/inattention, and compulsion and separation related behaviour. Finally, aggressive dogs were 3.2 times more often fearful, and dogs showing separation related behaviour were 2.8 times more likely fearful.

Comorbidity between subtraits was also common but showed high variability (Supplementary Table S3). Of the highly noise sensitive dogs, 53% displayed noise sensitivity toward more than one target. Similarly, 38% of the highly fearful dogs were highly fearful toward more than one subtrait. However, only 9% of aggressive dogs showed aggression toward both family members and strangers.

### Age and sex differences

**Figure 3:**
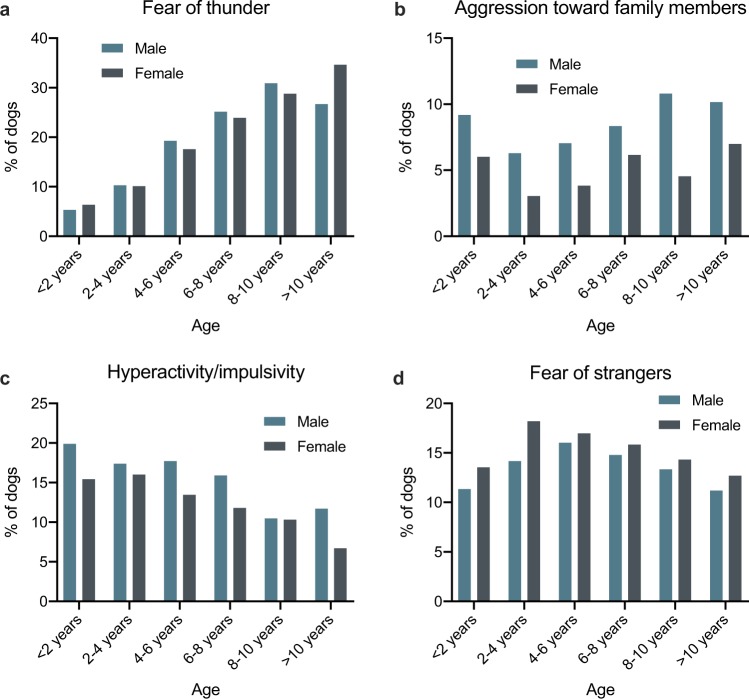
Prevalences of fear of thunder (**a**), aggression toward family members (**b**), hyperactivity/impulsivity (**c**) and fear of strangers (**d**) for both sexes and six age groups.

Male dogs were more often aggressive and hyperactive/impulsive, but female dogs were more often fearful (Fig. 3b–d, Supplementary Fig. S1, Supplementary Table S4). Separation related behaviour was slightly more common in male dogs. The prevalence of noise sensitivity increased with age, especially fear of thunder (Fig. 3a, Supplementary Fig. S1, Supplementary Table S5). Similarly, fear of surfaces and heights increased with age, whereas hyperactivity/impulsivity and tail chasing decreased. Other traits did not show clear linear changes.

### Breed specificity

**Figure 4:**
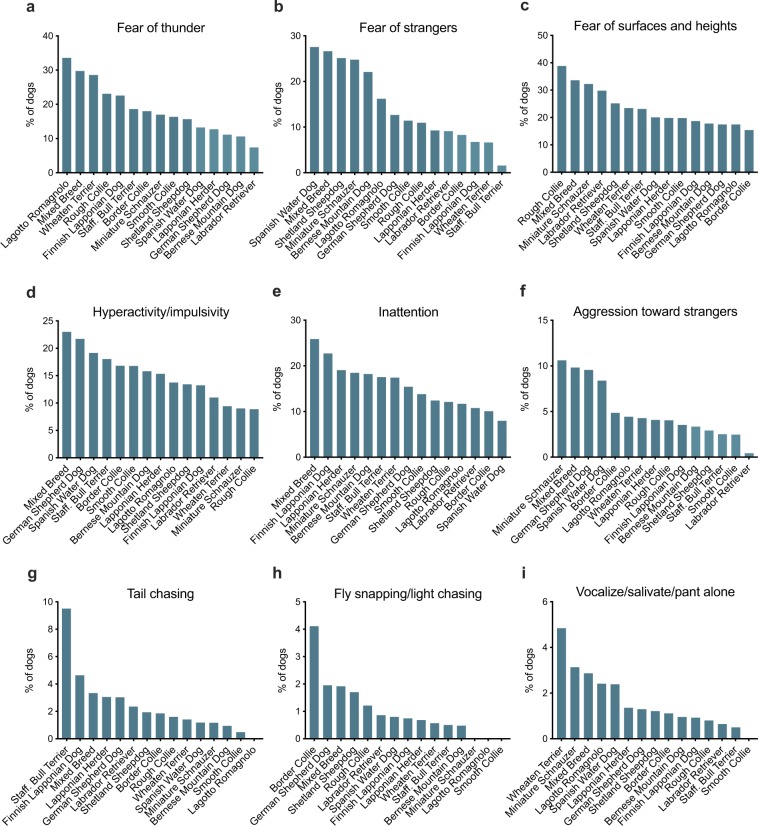
Breed differences in fear of thunder (**a**), fear of strangers (**b**), fear of surfaces and heights (**c**), hyperactivity/impulsivity (**d**), inattention (**e**), aggression toward strangers (**f**), tail chasing (**g**), fly snapping/light chasing (**h**) and vocalization/salivation/panting alone (**i**). For other breed-wise differences, see Supplementary Fig. S2.

Large breed differences were observed in all behavioural traits (Fig. 4, Supplementary Fig. S2, Supplementary Table S6). For example, 10.6% of Miniature Schnauzers were aggressive toward strangers, whereas only 0.4% of Labrador Retrievers showed aggression (Fig. 4). Similarly, 9.5% of Staffordshire Bull Terriers were reported to display tail chasing, but none of the Lagotto Romagnolo dogs chased their tails.

**Figure 5:**
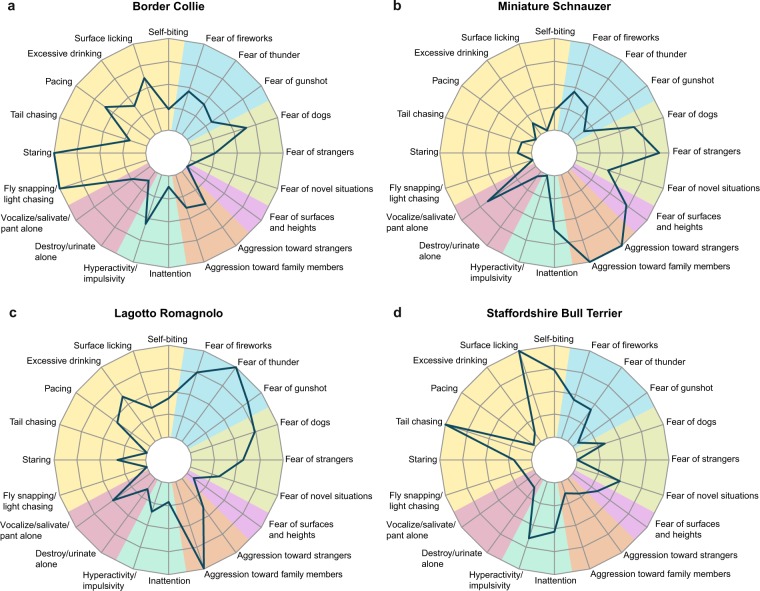
Radar chart representation of the behaviour of dog breeds: Border Collie (**a**), Miniature Schnauzer (**b**), Lagotto Romagnolo (**c**) and Staffordshire Bull Terrier (**d**). Colors represent the larger traits. Clockwise from top: blue – noise sensitivity, lime green – fear, violet – fear of surfaces and heights, orange – aggression, pine green – hyperactivity/inattention, purple – separation related behaviour, yellow – compulsive behaviour. Radar charts for other breeds in Supplementary Fig. S3. The minimum in all traits is the breed-wise minimum prevalence and the maximum in all traits is the breed-wise maximum prevalence. For the minimum and maximum prevalences, see Supplementary Table S7.

When focusing on individual breeds, these differences in prevalences resulted in breed-specific patterns (Fig. 5). Border Collies displayed a very high prevalence of compulsive staring and fly snapping, but only moderate levels of other traits (Fig. 5). In contrast, Miniature Schnauzers displayed high levels of aggression and social fear (fear of strangers and other dogs), but few stereotypies. Lagotto Romagnolos displayed high levels of noise sensitivity, social fear, and aggression. Staffordshire Bull Terriers showed high levels of compulsive behaviour, hyperactivity, and inattention. For more breeds, see Supplementary Fig. S3.

## Discussion

We have here developed a comprehensive behavioural survey and applied a citizen science approach to collect a large canine behavioural dataset of over 260 breeds of dogs. Our data replicates results from some earlier studies and the study also shows a number of novel insights, including prevalences, comorbidities and breed differences of traits not described before. These results improve the overall understanding of problematic behaviour.

Based on the results here and in previous studies, noise sensitivity stands out as the most common canine anxiety with a prevalence of 32% in this study. Earlier, the prevalence has varied between 20% and 50%^9,12,14–18^. Based on our study and previous studies^14,17,18^, the most common noise sensitivity is the fear of fireworks. Fear was the second most common canine anxiety, with a prevalence of 29%. Specifically, 17% of dogs showed fear of other dogs, 15% fear of strangers and 11% fear of novel situations. Prevalence of total fearfulness^12,16^ and prevalence of fear subtraits^9,15,16,35^ were quite similar in previous studies as well. Fear of surfaces and heights appears to be highly prevalent in our study population, as 23.5% of dog owners reported that their dogs were highly fearful of different surfaces and heights.

Based on our results, every fifth dog displays high levels of inattention and 15% high levels of hyperactivity/impulsivity. Excessive activity has been reported in 12% to 34% of dogs^9,12^. Compulsive behaviour patterns were observed in 16% of the dogs, agreeing with a previous study^9^. Based on our results and previous studies^9,28^, self-mutilation is the most common compulsive behaviour. Self-mutilation may be a compulsion, but it may also be caused by allergies, ectoparasites or other skin problems, possibly explaining the high prevalence of the subtrait.

The prevalence of aggression was 14%, with both aggression toward human family members and toward strangers occurring in 6% of dogs. In our previous study, 16% of dogs had at least once displayed aggressive behaviours toward family members and 45% toward strangers^16^. In previous studies, the prevalence of aggression and its subtraits has varied between 2% and 30%^9,12,15^. Studies focusing on referrals to veterinary/behaviour clinics often report aggression as the most common behaviour problem^10,11,13^, possibly because owners find aggressiveness more problematic than, for example, fear of fireworks. Separation related behaviours were only displayed by 6% of the dogs. Previously, the prevalence of separation anxiety has been 2–3 times higher than in this present study^9,15,16,19^, possibly because we only included dogs with high frequencies of separation related behaviour. Taken together, our results are surprisingly similar compared to previous studies, even though the populations studied and the criteria for anxiety-related traits differ in every study.

Male and female dogs displayed differences in the prevalence of behaviour problems. Male dogs had a higher prevalence of aggressiveness, separation related behaviour, inattention and hyperactivity/impulsivity. In contrast, female dogs had a higher prevalence of fearfulness. Noise sensitivity, fear of surfaces and compulsive behaviour occurred independent of sex. Previous studies have shown similar sex differences in aggression^9,13,36–38^, fearfulness^13,32,39–41^, separation related behaviour^36^ and compulsion^33^. Reported sex differences in noise sensitivity are less clear (more common in females^17,32^, more common in males^14^, no difference between sexes^18^).

We detected differences between age groups in the prevalence of most behaviour problems. Younger dogs had a higher prevalence of destroy/urinate when alone, inattention, hyperactivity/impulsivity, tail chasing and self-biting. Older dogs had a higher prevalence of aggression, noise sensitivity and fear of surfaces. Fearfulness was most common in dogs aged 4–8 years. Furthermore, vocalize/salivate/pant when alone and other compulsive behaviours (beside the aforementioned tail chasing and self-biting) occurred independent of age. Previous studies are in agreement at least in noise sensitivity^14,17,18,32^, inattention^42,43^, hyperactivity/impulsivity^12,42–44^ and aggression^12,37^. Tail chasing, destroying and urinating indoors are typical behaviours for puppies, and this likely explains the age differences in these subtraits.

We observed large behavioural differences between breeds. Noise sensitivity was the most common in Lagotto Romagnolo, Wheaten Terrier and mixed breed dogs. Previous studies have also ranked these breeds high in noise sensitivity^14,16–18^. Among some other breeds, Miniature Schnauzers and Staffordshire Bull Terriers were, in contrast, noise sensitive less often, as also observed in previous studies^10,17^. Fear was most common in Spanish Water Dogs, Shetland Sheepdogs and mixed breeds. In contrast, Labrador Retrievers were seldomly fearful. These results are in agreement with previous studies, ranking mixed breed dogs high in fearfulness^9,13,35,40^ and Labrador Retrievers and Staffordshire Bull Terriers low in fearfulness^35,41,45,46^. Fear of surfaces and heights was the most often observed in Rough Collie and mixed breed dogs.

Inattention was most often reported in mixed breed dogs, Finnish Lapponian Dogs and Lapponian Herders, and rarely reported in Spanish Water Dogs and Border Collies. Although Finnish Lapponian Dogs and Lapponian Herders have not been studied before, our results agree with previous results^43^. As the dog breeds showing high prevalence of inattention are breeds that are often regarded as “hard to train”, owners may rate dogs not easily motivated by food or petting as inattentive. Hyperactivity and impulsivity were the most common in mixed breed dogs, German Shepherds, Spanish Water Dogs and Staffordshire Bull Terriers, and the least common in Rough Collies and Miniature Schnauzers. Similar breed differences were observed in a previous study^44^. Furthermore, herding dogs (including, for example, Border Collie and German Shepherd) and terriers (including, for example, Staffordshire Bull Terriers) have ranked high in extraversion and, in contrast, toy dogs low in extraversion^47^. In our study, Labrador Retrievers and Rough Collies had a low prevalence of hyperactivity/impulsivity, but otherwise our results match previous studies. However, it seems that classification into traditional and genetic breed groups poorly reflect behavioural differences^48^, possibly explaining the differences in these results. Compulsive behaviour was most often reported by owners of German Shepherds, mixed breed dogs and Staffordshire Bull Terriers. However, the breed differences varied highly between different compulsions. For example, Staffordshire Bull Terriers had a high prevalence of tail chasing, with nearly 10% of them chasing their tails. In contrast, light chasing and staring were the most often observed in Border Collies. Interestingly, Border Collies were bred to herd livestock by staring at them intensely, and even though this method of herding can be perfected by training, the behaviour itself seems to be innate^49^. Pacing and excessive drinking were often performed by mixed breed dogs and German Shepherds. In a previous study, German shepherds had high odds of being presented to a behaviour clinic for obsessive behaviour^10^.

Mixed breed dogs and Miniature Schnauzers had the highest prevalence of aggression, whereas Labrador Retrievers had the lowest prevalence of aggression. Aggression toward strangers was most prevalent in Miniature Schnauzers, mixed breed dogs, German Shepherd Dogs and Spanish Water Dogs, and least prevalent in Labrador Retrievers. Aggression toward human family members was most common in Miniature Schnauzers and Lagotto Romagnolos. Our results agree with previous studies both in total aggression^9,13^ and in the subtraits, aggression toward strangers^36–38,45^ and aggression towards family members^16,37^. Separation related behaviour was most common in mixed breed dogs and Wheaten Terriers. Specifically, mixed breed dogs were likely to destroy, urinate or defecate when left alone, whereas Wheaten Terriers were likely to vocalize, salivate or pant. Based on our results and a previous study^36^, mixed breed dogs may be more prone to show separation related behaviour. It is possible that the high prevalence of separation distress and other anxieties in the mixed breed dogs is caused by a poor early life environment and adverse experiences in life, as many mixed breed dogs in our data are likely rescues.

Within-trait comorbidity was common in noise sensitivity and fear: 53% of dogs that were fearful of one noise were fearful of several noises, and 38% of fearful dogs were fearful of more than one target. This result was also discovered in our previous study^16^. Based on previous studies, noise sensitivity is often generalised and displayed toward several different noises^9,14,17,18^. We discovered that dogs were seldomly aggressive toward both family members and strangers, as reported before in some dog breeds^50^. In contrast, one previous study did report a significant comorbidity between stranger-directed and owner-directed aggression^13^. However, it seems that aggression toward strangers and family members are genetically distinct traits^51^.

We discovered that noise sensitivity and fear were the most common comorbidities, likely due to their high prevalence in our study population. However, when comparing the risk ratios in comorbid traits, the largest risk ratios were seen between separation related behaviour, hyperactivity/impulsivity, inattention and compulsive behaviour, and between fear and aggression. Fearful dogs were 3.2 times more often aggressive than non-fearful dogs, a relationship found in previous studies as well^9,13,16,38^. This indicates that aggression is commonly motivated by fear. The connection between impulsivity, compulsive behaviour and separation related behaviour is an interesting finding that demands further research. One previous study discovered that excitable dogs had 9.8 times higher odds of separation distress^52^ and another study discovered a connection between compulsive behaviour and hyperactivity^9^. Intriguingly, impulsivity and compulsion are related constructs, as both are proposed to be caused by a failure of response control and mediated by basal ganglia^53^. We observed many trait connections detected in previous studies as well, including comorbidity between fear and noise sensitivity^14,16,17^, between fear and separation related behaviour^16^, between separation related behaviour and aggression^13,16^ and between fear and compulsive behaviour^9,33^. However, previous studies have detected a comorbidity between separation anxiety and noise sensitivity^9,13,15,17,19,20^. We indeed discovered that separation related behaviour was 1.4 times more prevalent in noise sensitive dogs. However, the opposite was not true, as dogs showing separation related behaviour were not fearful of noises more often than dogs not showing separation related behaviour. Furthermore, we discovered a positive connection between compulsive behaviour and aggression, contrasting with the results of our previous study^33^.

This study has limitations. Although the fear section of the questionnaire was validated and the test-retest reliability of the fear and noise sensitivity sections was good^54^ and that the results we have obtained from the data collected with it^32,33,55^ replicate many previous results, the psychometric properties of the rest of the questionnaire have not been formally evaluated. Future studies should aim to assess the reliability and validity of these additional components. Secondly, the categorisation into low, moderate, and high categories was mostly based on the frequency of signs and not the severity, except in aggression, separation anxiety, and impulsivity/inattention. Thus, in the high groups, the severity of the symptoms can be variable. Thirdly, our sample is a self-selected convenience sample, and may not be representative of the overall Finnish dog population. Although the most common breeds in our sample are also common in Finland^56^, the representativeness of our sample is still unknown. We are currently working on a separate study to understand the participant profiles and details of the sample demographics.

Our findings on breed differences indicate that canine anxieties likely have a genetic basis. In previous studies, many behavioural traits have been indeed shown to have small to moderate heritabilities^22,26,57^ and recently we mapped two loci for generalized fear and noise sensitivity^30^. Therefore, it could be possible to decrease the prevalence of canine anxieties by selecting non-anxious animals for breeding. Our results also show that these canine anxieties are phenotypically correlated. Some of these traits, like many behaviour traits^21,22^, may also be genetically correlated, and therefore selection for one trait may influence other traits as well. Interestingly, a genomic region associated with noise sensitivity in German Shepherd Dogs^30^ contains the oxytocin receptor gene (OXTR). The gene is associated with social behaviour^58^ (but see a contrasting study with a smaller sample size^59^) and most likely has been under strong selection during domestication. This could explain the high prevalence of noise sensitivity in many study populations^12,14–18^ and could also indicate that breeding efforts to reduce the prevalence of noise sensitivity may prove difficult.

Based on our results, canine anxieties and behaviour problems are common across breeds. There are around 77 million dogs in the United States^60^ and 85 million in Europe^61^, and therefore these behaviour problems can affect millions of animals. As anxiety can impair welfare^1^ and problematic behaviour may be an indication of poor welfare^62^, efforts should be made to decrease the prevalence of these canine anxieties. Breeding policies may help to improve dog welfare, as could changes in the living environment^14,19,32,33,37^. Our ongoing efforts aim to identify environmental and genetic risk factors behind these canine anxiety-related traits using the large survey data collected here.

## Methods

### Questionnaire

A comprehensive online questionnaire was designed to collect extensive information about behaviour and background information of dogs (Supplementary information). The questionnaire consisted of a background section and sections focusing on seven canine anxiety-related traits: noise sensitivity, fear, fear of surfaces and heights, impulsivity/inattention, compulsive behaviour, aggression and separation related behaviour. These traits consisted of several subtraits (Fig. 1b). The fear part of this questionnaire was previously shown to have good validity and both the fear and noise sensitivity sections had good test-retest reliability^54^. A questionnaire can be a good method for collecting data, since the reliability of questionnaires has been good in behavioural science and the answers are strongly linked to the behaviour of the animals^54,63–68^. Furthermore, as the owners have a long history with the dogs, it is possible to study traits that would be difficult to study using other methods (for example, behaviour test batteries), including compulsive behaviour^69^.

### Subjects

Owners were mainly recruited from a social media (Facebook) channel. Furthermore, dog breed organisations advertised the questionnaire on their own websites and Facebook pages. In total, we received questionnaire responses of 13,715 dogs in 264 breeds. To ensure reliable prevalence estimates in breed comparisons, we calculated a minimum sample size per breed with the Epitools sample size calculator^70^. For this calculation, we used the average prevalence of behaviour traits (19%), the average number of registrations per dog breed within the last 10 years (1500), a desired precision of 0.05 and a confidence level of 0.95. This calculation resulted in a minimum sample size of 200 individuals per breed. We obtained more than 200 answers from 14 breeds and mixed breed dogs (N = 418): Finnish Lapponian Dog (N = 538), Labrador Retriever (N = 465), German Shepherd Dog (N = 461), Shetland Sheepdog (N = 411), Soft-coated Wheaten Terrier (N = 351), Lapponian Herder (N = 294), Border Collie (N = 268), Miniature Schnauzer (N = 255), Spanish Water Dog (N = 251), Rough Collie (N = 248), Lagotto Romagnolo (N = 248), Bernese Mountain Dog (N = 209), Smooth Collie (N = 203) and Staffordshire Bull Terrier (N = 200).

Informed consent was obtained from all participants. Participants agreed that all questionnaire answers could be used for research. We emphasized that all data will be handled strictly confidentially, and that individual dogs and owners cannot be recognized from the published results.

### Subject categorisation

Based on the questionnaire scores, dogs were categorised into low, moderate, and high groups for each subtrait (Fig. 1b, Supplementary Table S1) depending on the frequency of the behaviour. The subtraits were, in turn, combined to form trait groups. Low trait groups consisted of dogs that fell into low groups in all subtraits, and high trait groups consisted of dogs that had a high score in at least one subtrait. The categorisation for different subtraits is explained below.

#### Noise sensitivity

Noise sensitivity consisted of three subtraits: fear of thunder, fireworks, and gunshot. The respondents were first asked whether their dog showed fear toward these targets, and secondly, how often their dog shows fear, from rarely (0–20% of the time) to always (100% of the time). Dogs belonging to low group did not show fear toward these targets and the owners did not report a frequency for noise sensitivity. Dogs belonging to the high group showed fear at least often (40–60% of the time).

#### Fear

The fear section of the questionnaire consisted of three fear subtraits: fear of strangers, other dogs, and novel situations. Dogs that showed fear at least often (40–60% of the time) constituted the high group and dogs that never showed fear constituted the low group in all subtraits. Furthermore, low fear dogs never barked or growled at strangers or other dogs. Behaviour tests have previously been conducted to validate fear of strangers and fear of novel situations phenotypes^54^.

#### Fear of surfaces and heights

In this section, owners were asked whether their dog had difficulties walking on different surfaces, including on a metal grid, on shiny floors, or moving from one surface to another. Moreover, owners were asked whether their dog had difficulties in high places: climbing stairs where you can or cannot see between steps, walking next to glass railings, climbing metal stairs, and walking over narrow bridges. In all questions, these answers were scored between 0 (never) and 4 (always). Dogs were categorised as high fear of surfaces if at least one of the answers was 3 (often) or 4, and low group consisted of dogs that never had difficulties in any of these tasks.

#### Impulsivity/inattention

Dog owners were asked to rate their dog’s behaviour on a scale of 1 (never) to 4 (very often) in 13 questions, developed by Vas and colleagues^42^ and designed to measure inattention, hyperactivity, and impulsivity. A principal component analysis with a promax rotation was run to divide the questions into two components, inattention and hyperactivity/impulsivity (Supplementary Table S8), and component scores were calculated for each dog. One question (Item 11) was removed from the analysis, as it loaded equally on both components. The cut-off between low and moderate category was set at the first dog having a rating of 3 (often) in the item that had the highest loading (Item 2 in inattention, Item 5 in hyperactivity/impulsivity). The cut-off between the moderate and high category was set at the first dog having a rating of 4 (very often) in the highest loading item and the same rating on one additional item.

#### Compulsive behaviour

Compulsive behaviour part of the questionnaire measured the occurrence of tail chasing, fly snapping/light chasing, surface licking, pacing, staring, excessive drinking, and self-biting. Tail chasing, fly snapping/light chasing, surface licking, pacing, and staring were scored on a scale of 0 (I’ve never noticed this behaviour) to 6 (several times per day). The dogs scoring from 4 (every other day-weekly) to 6 were placed in the high group, and dogs scoring 0 or 1 (a few times during the dog’s lifetime) were placed in the low group. Furthermore, owners were asked to estimate the time dog spent drinking or near the water bowl from less than 5 minutes to 1 hour or more. Dogs that spent less than 5 minutes near the water bowl formed the low group in excessive drinking, and dogs spending more than 15 minutes near it formed the high group. Finally, the prevalence of self-biting was rated from 0 (never) to 3 (several hours per day). Dogs scoring 2 (almost every day) or 3 constituted the high group whereas dogs scoring 0 constituted the low group.

#### Aggression

The aggression trait in the questionnaire consisted of two subtraits: aggression toward strangers and toward family members. The respondents were asked to score the likelihood of their dog growling and trying to snap/bite from 1 (never) to 5 (always or almost always) when a stranger tries to pet the dog in its home or outside and when the owner handles the dog or tries to take a resource (food, bone, or toy) from the dog. If the dog tried to snap or bite at least sometimes (3) or it growled at least often (4), it was categorised as a high aggression dog in each subtrait. The dogs that never showed aggression in any of these situations constituted the low group.

#### Separation related behaviour

Separation related behaviour was divided into two parts: destroy/urinate alone and vocalize/salivate/pant alone. Dog owners were asked whether their dog exhibited separation anxiety and how often did the dog perform the aforementioned behaviours from 0 (never) to 4 (very often). High group dogs were reported to exhibit separation anxiety and performed at least one of these behaviours often (3) or very often. With the subtrait vocalize/salivate/pant, high group dogs were reported to salivate or pant at least often (3–4), or barked at least often (3–4) and salivated or panted at least rarely (1–4). Low group dogs did not show separation anxiety and never performed these behaviours.

### Statistical analyses

To estimate the prevalence of high anxiety dogs, the number of dogs in high group was divided by the sum of dogs in all trait groups, hence obtaining the percentage of dogs in the high group for each subtrait and trait in the population.

The same prevalence calculation was performed individually for different breeds (sample size > 200) and for defined age groups and both sexes. The dogs were divided into six age groups: less than 2 years, 2–4 years, 4–6 years, 6–8 years, 8–10 years, and more than 10 years. Chi-squared tests were run in R^71^ for breed, sex, and age group differences in all traits and subtraits to reveal the significant associations.

Comorbidities were calculated between all pairs of behaviour problems. In the pairs comorbidity/diagnosis adapted from Goldstein-Piekarsi *et al*.^72^, the number of dogs belonging to the high group in both of the traits was divided by the number of all dogs in the high group in the denominator trait, hence obtaining the percentage of dogs in each denominator trait that also had the comorbid trait. The relative risk was calculated by firstly calculating the same percentage and secondly calculating the percentage of dogs that belonged to the low group in the denominator trait but in the high group in the other trait. Thirdly, the first percentage was divided by the second. Two-proportions z-tests were run in R^71^ for all risk ratios to see whether any two traits have statistically significant comorbidity.

All P-values were corrected for false discovery rate (FDR) to decrease the probability of type I error. The significance cut-off P-value was set at P < 0.05.

## Supplementary information

Supplementary Information.

Dataset 1.

## Data Availability

The anonymised data is available as a supplementary datasheet.
