# Supplementary material for: Prevalence, comorbidity, and breed differences in canine anxiety in 13,700 Finnish pet dogs
Source: Sci Rep. 2020 Mar 5;10:2962. doi: 10.1038/s41598-020-59837-z (PMC7058607; doi:10.1038/s41598-020-59837-z)
Supplement: Supplementary file 1 — Supplementary Information. [file 41598_2020_59837_MOESM1_ESM.pdf]

Prevalence, comorbidity, and breed differences in canine anxiety in 13,700 Finnish pet dogs

Milla Salonen[1,2,3], Sini Sulkama[1,2,3], Salla Mikkola[1,2,3], Jenni Puurunen[1,2,3], Emma Hakanen[1,2,3], Katriina Tiira[1,2,3], César Araujo[1,2,3], Hannes Lohi[1,2,3*]

[1] Department of Medical and Clinical Genetics, University of Helsinki, Helsinki, Finland

[2] Department of Veterinary Biosciences, University of Helsinki, Helsinki, Finland

[3] Folkhälsan Research Center, Helsinki, Finland

[*] Corresponding author:

Hannes Lohi, PhD, Professor

Email: hannes.lohi@helsinki.fi (HL)

Supplementary tables

Supplementary Table S1. Behaviour trait and subtrait prevalences in the sample of 13,715 Finnish pet dogs.

Behaviour trait	Behaviour subtrait	Percentage (count in parentheses)	
Noise sensitivity		32.4%	(4,440)
	Fear of fireworks	25.8%	(3,543)
	Fear of thunder	16.7%	(2,285)
	Fear of gunshot	12.7%	(1,743)
Fear		28.6%	(3,929)
	Fear of dogs	16.5%	(2,266)
	Fear of strangers	14.7%	(2,015)
	Fear of novel situations	11.3%	(1,544)
Fear of surfaces and heights		23.5%	(3,227)
Inattention		19.1%	(2,617)
Hyperactivity/impulsivity		15.0%	(2,052)
Compulsive behaviour		16.0%	(2,190)
	Self-biting	6.3%	(863)
	Surface licking	4.6%	(632)
	Excessive drinking	2.5%	(348)
	Tail chasing	2.4%	(324)
	Pacing	2.1%	(287)
	Fly snapping/light chasing	1.2%	(168)
	Staring	0.9%	(126)
Aggression		14.2%	(1,946)
	Aggression toward family members	6.4%	(883)
	Aggression toward strangers	5.9%	(815)
Separation related behaviour		5.5%	(748)
	Destroy/urinate alone	3.7%	(508)
	Vocalize/salivate/pant alone	2.5%	(340)

Supplementary Table S2. Relative risk of comorbidities. DF = 1 in all comparisons.

	Noise sensitivity	Fearfulness	Aggression	Fear of surfaces	Compulsive behavior	Separation anxiety	Inattention	Hyperactivity/ impulsivity
Hyperactivity/ impulsivity	RR = 1.27<br>$\chi^2 = 40.88$<br>P-value < 0.001	RR = 2.27<br>$\chi^2 = 443.69$<br>P-value < 0.001	RR = 2.30<br>$\chi^2 = 188.45$<br>P-value < 0.001	RR = 1.34<br>$\chi^2 = 38.92$<br>P-value < 0.001	RR = 3.19<br>$\chi^2 = 434.27$<br>P-value < 0.001	RR = 4.09<br>$\chi^2 = 202.37$<br>P-value < 0.001		
Inattention	RR = 1.28<br>$\chi^2 = 41.16$<br>P-value < 0.001	RR = 1.93<br>$\chi^2 = 258.67$<br>P-value < 0.001	RR = 1.95<br>$\chi^2 = 106.78$<br>P-value < 0.001	RR = 1.67<br>$\chi^2 = 123.53$<br>P-value < 0.001	RR = 2.39<br>$\chi^2 = 219.75$<br>P-value < 0.001	RR = 3.42<br>$\chi^2 = 126.17$<br>P-value < 0.001		
Separation anxiety	RR = 1.04<br>$\chi^2 = 0.56$<br>P-value = 0.483	RR = 2.00<br>$\chi^2 = 197.32$<br>P-value < 0.001	RR = 2.01<br>$\chi^2 = 77.83$<br>P-value < 0.001	RR = 1.30<br>$\chi^2 = 16.28$<br>P-value < 0.001	RR = 2.53<br>$\chi^2 = 182.45$<br>P-value < 0.001		RR = 2.48<br>$\chi^2 = 223.00$<br>P-value < 0.001	RR = 3.26<br>$\chi^2 = 304.91$<br>P-value < 0.001
Compulsive behavior	RR = 1.24<br>$\chi^2 = 35.79$<br>P-value < 0.001	RR = 1.60<br>$\chi^2 = 153.20$<br>P-value < 0.001	RR = 1.93<br>$\chi^2 = 130.59$<br>P-value < 0.001	RR = 1.60<br>$\chi^2 = 112.20$<br>P-value < 0.001		RR = 2.80<br>$\chi^2 = 119.33$<br>P-value < 0.001	RR = 2.52<br>$\chi^2 = 357.10$<br>P-value < 0.001	RR = 3.06<br>$\chi^2 = 413.77$<br>P-value < 0.001
Fear of surfaces	RR = 1.41<br>$\chi^2 = 73.39$<br>P-value < 0.001	RR = 1.95<br>$\chi^2 = 221.42$<br>P-value < 0.001	RR = 1.71<br>$\chi^2 = 52.48$<br>P-value < 0.001		RR = 1.82<br>$\chi^2 = 77.36$<br>P-value < 0.001	RR = 1.69<br>$\chi^2 = 16.88$<br>P-value < 0.001	RR = 2.21<br>$\chi^2 = 154.42$<br>P-value < 0.001	RR = 1.61<br>$\chi^2 = 43.60$<br>P-value < 0.001
Aggression	RR = 1.34<br>$\chi^2 = 68.97$<br>P-value < 0.001	RR = 2.46<br>$\chi^2 = 723.89$<br>P-value < 0.001		RR = 1.39<br>$\chi^2 = 60.01$<br>P-value < 0.001	RR = 1.80<br>$\chi^2 = 136.29$<br>P-value < 0.001	RR = 1.91<br>$\chi^2 = 51.67$<br>P-value < 0.001	RR = 1.75<br>$\chi^2 = 143.65$<br>P-value < 0.001	RR = 1.96<br>$\chi^2 = 169.24$<br>P-value < 0.001
Fearfulness	RR = 1.52<br>$\chi^2 = 202.42$<br>P-value < 0.001		RR = 3.21<br>$\chi^2 = 558.83$<br>P-value < 0.001	RR = 1.94<br>$\chi^2 = 335.23$<br>P-value < 0.001	RR = 1.75<br>$\chi^2 = 148.18$<br>P-value < 0.001	RR = 2.79<br>$\chi^2= 151.15$<br>P-value < 0.001	RR = 2.00<br>$\chi^2 = 280.99$<br>P-value < 0.001	RR = 1.96<br>$\chi^2 = 354.91$<br>P-value < 0.001
Noise sensitivity		RR = 1.68<br>$\chi^2 = 233.08$<br>P-value < 0.001	RR = 1.43<br>$\chi^2 = 47.18$<br>P-value < 0.001	RR = 1.69<br>$\chi^2 = 179.86$<br>P-value < 0.001	RR = 1.53<br>$\chi^2 = 69.95$<br>P-value < 0.001	RR = 1.44<br>$\chi^2 = 14.85$<br>P-value < 0.001	RR = 1.44<br>$\chi^2 = 243.49$<br>P-value < 0.001	RR = 1.44<br>$\chi^2 = 49.90$<br>P-value < 0.001

Supplementary Table S3. Within-trait comorbidity of the anxiety-like behaviour traits

Trait	Percentage (count in parentheses) Comorbidity		No comorbidity	
Noise sensitivity	52.5%	(2,330)	47.5%	(2,110)
Fear	38.2%	(1,499)	61.8%	(2,430)
Compulsion	19.2%	(420)	80.8%	(1,770)
Separation anxiety	13.4%	(100)	86.6%	(648)
Aggression	8.7%	(169)	91.3%	(1,777)

Supplementary Table S4. Chi square test of sex differences in behaviour traits and subtraits.

DF = 1. P values that are significant after FDR correction are bolded.

**Behaviour trait**	**Behaviour subtrait**	χ^2	**P-value**
Noise sensitivity		0.44	0.537
	Fear of fireworks	0.001	0.978
	Fear of thunder	1.23	0.290
	Fear of gunshot	3.37	0.078
Fear		8.20	**0.005**
	Fear of dogs	21.31	**<0.001**
	Fear of strangers	8.48	**0.005**
	Fear of novel situations	0.36	0.57
Fear of surfaces and heights		2.54	0.126
Inattention		42.61	**<0.001**
Hyperactivity/impulsivity		23.14	**<0.001**
Compulsive behaviour		4.98	**0.031**
	Self-biting	0.39	0.553
	Surface licking	2.64	0.121
	Excessive drinking	1.88	0.187
	Tail chasing	0.25	0.630
	Pacing	1.97	0.179
	Fly snapping/light chasing	0.89	0.372
	Staring	1.54	0.235
Aggression		39.01	**<0.001**
	Aggression toward family members	54.46	**<0.001**
	Aggression toward strangers	12.71	**<0.001**
Separation related behaviour		21.60	**<0.001**
	Destroy/urinate alone	8.25	**0.005**
	Vocalize/salivate/pant alone	15.18	**<0.001**

Supplementary Table S5. Chi square test of age differences in behaviour traits and subtraits. DF = 5. P values that are significant after FDR correction are bolded.

**Behaviour trait**	**Behaviour subtrait**	χ^2	**P-value**
Noise sensitivity		323.53	**<0.001**
	Fear of fireworks	373.76	**<0.001**
	Fear of thunder	577.09	**<0.001**
	Fear of gunshot	287.39	**<0.001**
Fear		32.29	**<0.001**
	Fear of dogs	37.50	**<0.001**
	Fear of strangers	26.02	**<0.001**
	Fear of novel situations	24.23	**<0.001**
Fear of surfaces and heights		76.30	**<0.001**
Inattention		27.05	**<0.001**
Hyperactivity/impulsivity		66.63	**<0.001**
Compulsive behaviour		34.70	**<0.001**
	Self-biting	17.07	**0.005**
	Surface licking	9.02	0.124
	Excessive drinking	8.23	0.161
	Tail chasing	211.42	**<0.001**
	Pacing	12.80	**0.030**
	Fly snapping/light chasing	8.25	0.161
	Staring	2.64	0.767
Aggression		38.19	**<0.001**
	Aggression toward family members	37.51	**<0.001**
	Aggression toward strangers	33.86	**<0.001**
Separation related behaviour		40.11	**<0.001**
	Destroy/urinate alone	69.01	**<0.001**
	Vocalize/salivate/pant alone	4.25	0.538

Supplementary Table S6. Chi square test of breed differences in behaviour traits and subtraits.

DF = 14. P values that are significant after FDR correction are bolded.

**Behaviour trait**	**Behaviour subtrait**	χ^2	**P-value**
Noise sensitivity		78.253	**<0.001**
	Fear of fireworks	136.83	**<0.001**
	Fear of thunder	124.45	**<0.001**
	Fear of gunshot	102.33	**<0.001**
Fear		99.399	**<0.001**
	Fear of dogs	124.53	**<0.001**
	Fear of strangers	187.81	**<0.001**
	Fear of novel situations	58.878	**<0.001**
Fear of surfaces and heights		70.356	**<0.001**
Inattention		65.746	**<0.001**
Hyperactivity/impulsivity		54.289	**<0.001**
Compulsive behaviour		57.014	**<0.001**
	Self-biting	45.13	**<0.001**
	Surface licking	25.249	**0.038**
	Excessive drinking	36.914	**<0.001**
	Tail chasing	64.205	**<0.001**
	Pacing	36.733	**0.001**
	Fly snapping/light chasing	38.799	**<0.001**
	Staring	27.473	**0.020**
Aggression		105.82	**<0.001**
	Aggression toward family members	74.027	**<0.001**
	Aggression toward strangers	86.201	**<0.001**
Separation related behaviour		41.284	**<0.001**
	Destroy/urinate alone	44.089	**<0.001**
	Vocalize/salivate/pant alone	41.627	**<0.001**

Supplementary Table S7. Minimum and maximum breed-wise prevalences in behaviour traits and subtraits. These minimum and maximum prevalences were used as minimum and maximum values in the radar charts (Figure 5 and Supplementary Figure S3).

**Behaviour trait/subtrait**	**Minimum prevalence**	**Maximum prevalence**
Noise sensitivity	19.7% (German Shepherd Dog)	50.2% (Mixed Breed)
Fear of fireworks	11.0% (Labrador Retriever)	44.0% (Mixed Breed)
Fear of thunder	7.3% (Labrador Retriever)	33.5% (Lagotto Romagnolo)
Fear of gunshot	6.2% (Bernese Mountain Dog)	24.7% (Labrador Retriever)
Fear	17.6% (Labrador Retriever)	44.5 % (Mixed Breed)
Fear of dogs	3.3% (Bernese Mountain Dog)	26.7% (Spanish Water Dog)
Fear of strangers	1.5% (Staffordshire Bull Terrier)	27.5% (Spanish Water Dog)
Fear of novel situations	5.4% (Labrador Retriever)	19.6% (Mixed Breed)
Fear of surfaces and heights	15.3% (Border Collie)	38.7% (Rough Collie)
Inattention	8.0% (Spanish Water Dog)	25.8% (Mixed Breed)
Hyperactivity/impulsivity	8.9% (Rough Collie)	23.0% (Mixed Breed)
Compulsive behaviour	7.8% (Miniature Schnauzer)	23.0% (German Shepherd Dog)
Self-biting	3.0% (Smooth Collie)	11.2% (Mixed Breed)
Surface licking	1.1% (Wheaten Terrier)	6.0% (Staffordshire Bull Terrier)
Excessive drinking	0.5% (Smooth Collie)	5.0% (Mixed Breed)
Tail chasing	0.0% (Lagotto Romagnolo)	9.5% (Staffordshire Bull Terrier)
Pacing	0.4% (Miniature Schnauzer)	4.1% (Mixed Breed)
Fly snapping/ light chasing	0.0% (Lagotto Romagnolo, Miniature Schnauzer & Smooth Collie)	4.1% (Border Collie)
Staring	0.0% (Bernese Mountain Dog & Shetland Sheepdog)	2.6% (Border Collie)
Aggression	4.1% (Labrador Retriever)	24.4% (Mixed Breed)
Aggression toward family members	1.0% (Lapponian Herder)	12.2% (Miniature Schnauzer)
Aggression toward strangers	0.4% (Labrador Retriever)	10.6% (Miniature Schnauzer)
Separation related behaviour	1.5% (Staffordshire Bull Terrier)	8.1% (Mixed Breed)
Destroy/urinate alone	0.8% (Rough Collie)	6.5% (Mixed Breed)
Vocalize/salivate/ pant alone	0.0% (Smooth Collie)	4.8% (Wheaten Terrier)

Supplementary Table S8. Component loadings of questionnaire items in inattention and hyperactivity/impulsivity traits. Item 2 had the highest loading for inattention and was used in determining the cut-off points for that trait. Similarly, Item 5 had the highest loading for hyperactivity/impulsivity. For the questionnaire item descriptions, see Vas J, Topál J, Péch É, Miklósi Á. Measuring attention deficit and activity in dogs: A new application and validation of a human ADHD questionnaire. *Appl Anim Behav Sci.* 2007;103(1):105–17.

	Inattention	Hyperactivity/ impulsivity
Item 1	**0.71**	0.16
Item 2	**0.96**	-0.36
Item 3	**0.85**	-0.02
Item 4	**0.44**	0.26
Item 5	-0.12	**0.86**
Item 6	-0.06	**0.83**
Item 7	**0.56**	0.14
Item 8	0.07	**0.79**
Item 9	-0.17	**0.75**
Item 10	**0.65**	0.06
Item 12	**0.65**	0.23
Item 13	0.24	**0.64**

Supplementary figures

Supplementary Figure S1. Prevalences of behaviour traits for both sexes and six age groups.

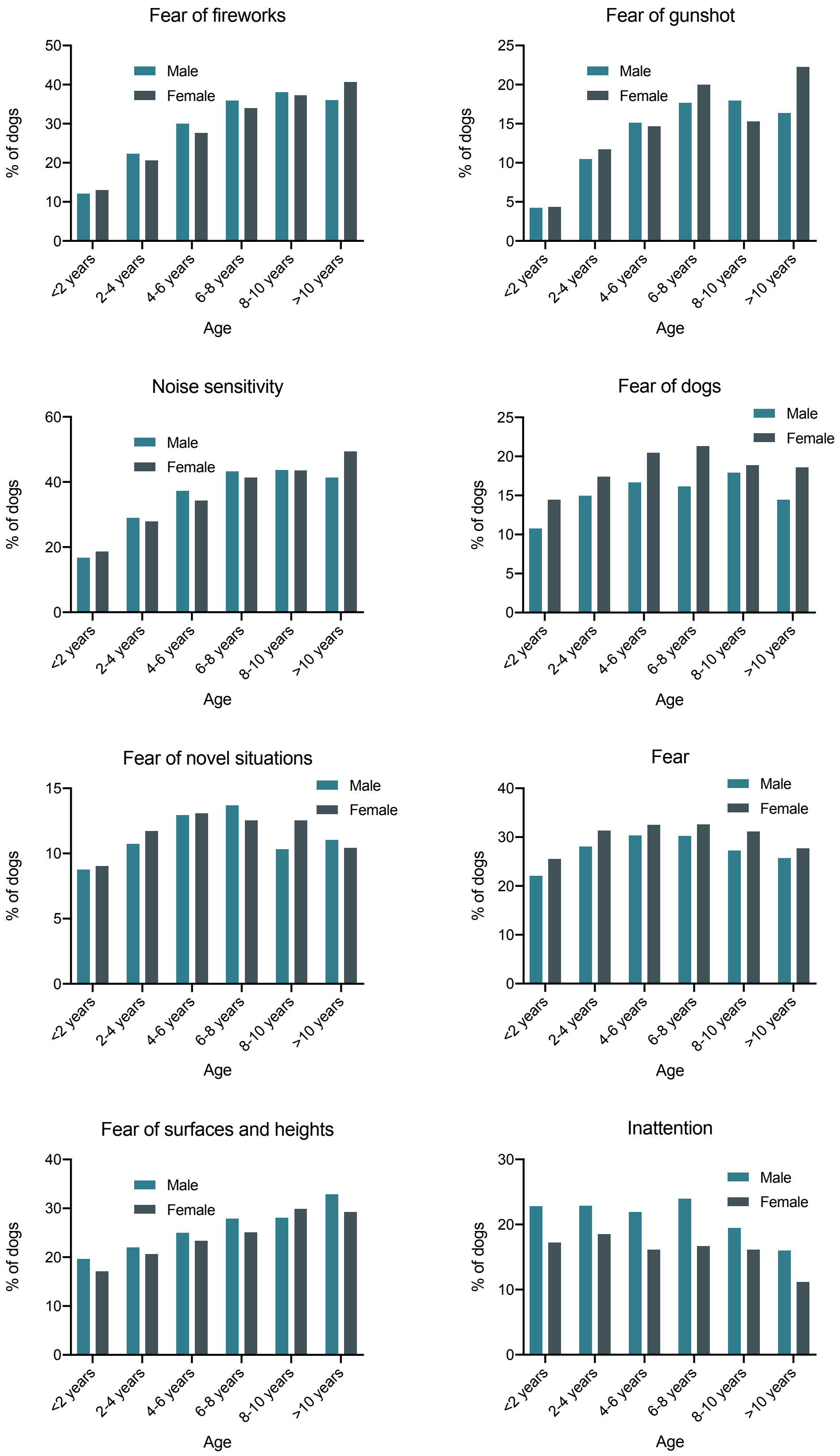

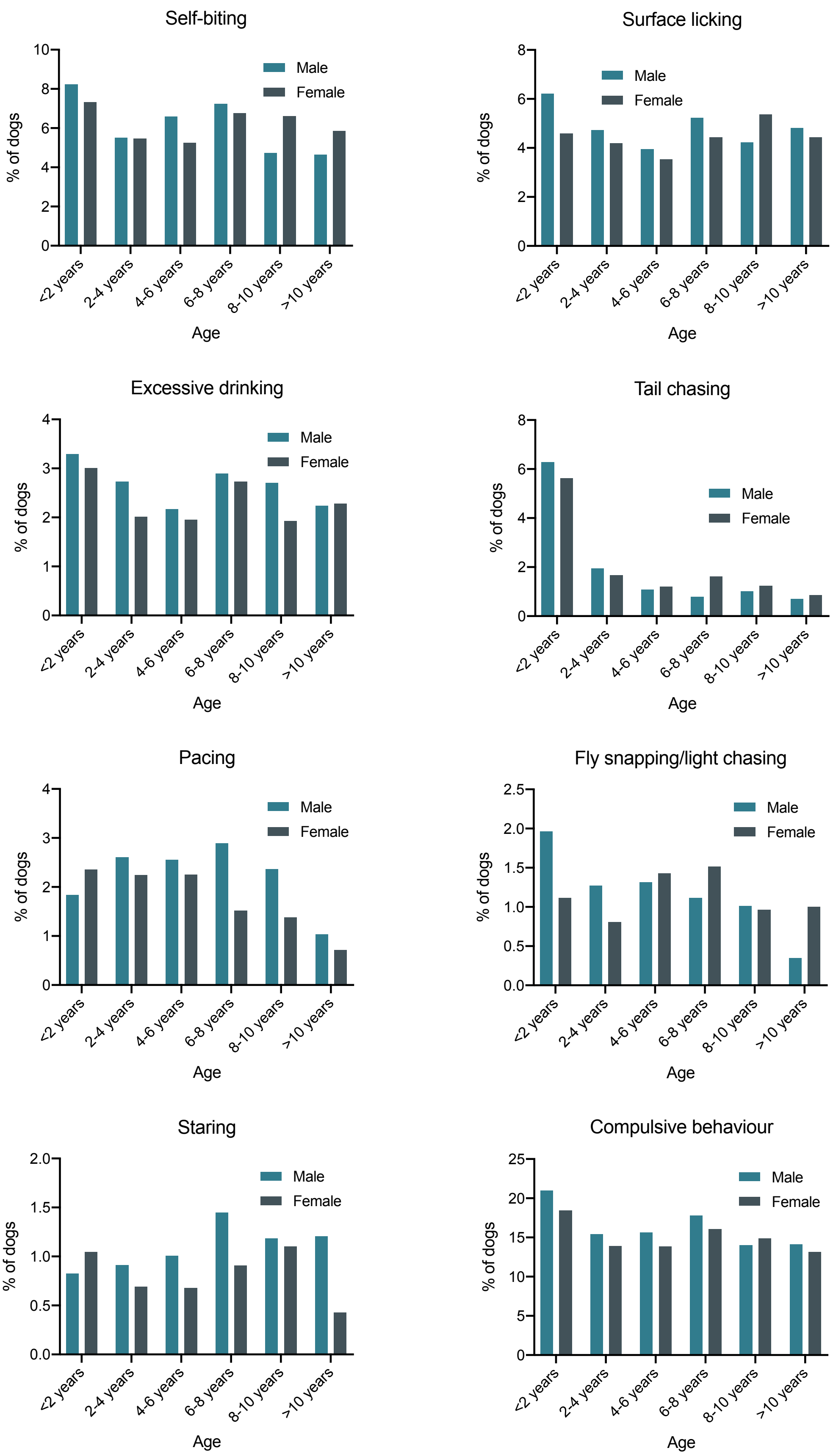

Self-biting
Surface licking
Excessive drinking
Tail chasing
Pacing
Fly snapping/light chasing
Staring
Compulsive behaviour
Male
Female
% of dogs
Age
<2 years
2-4 years
4-6 years
6-8 years
8-10 years
>10 years

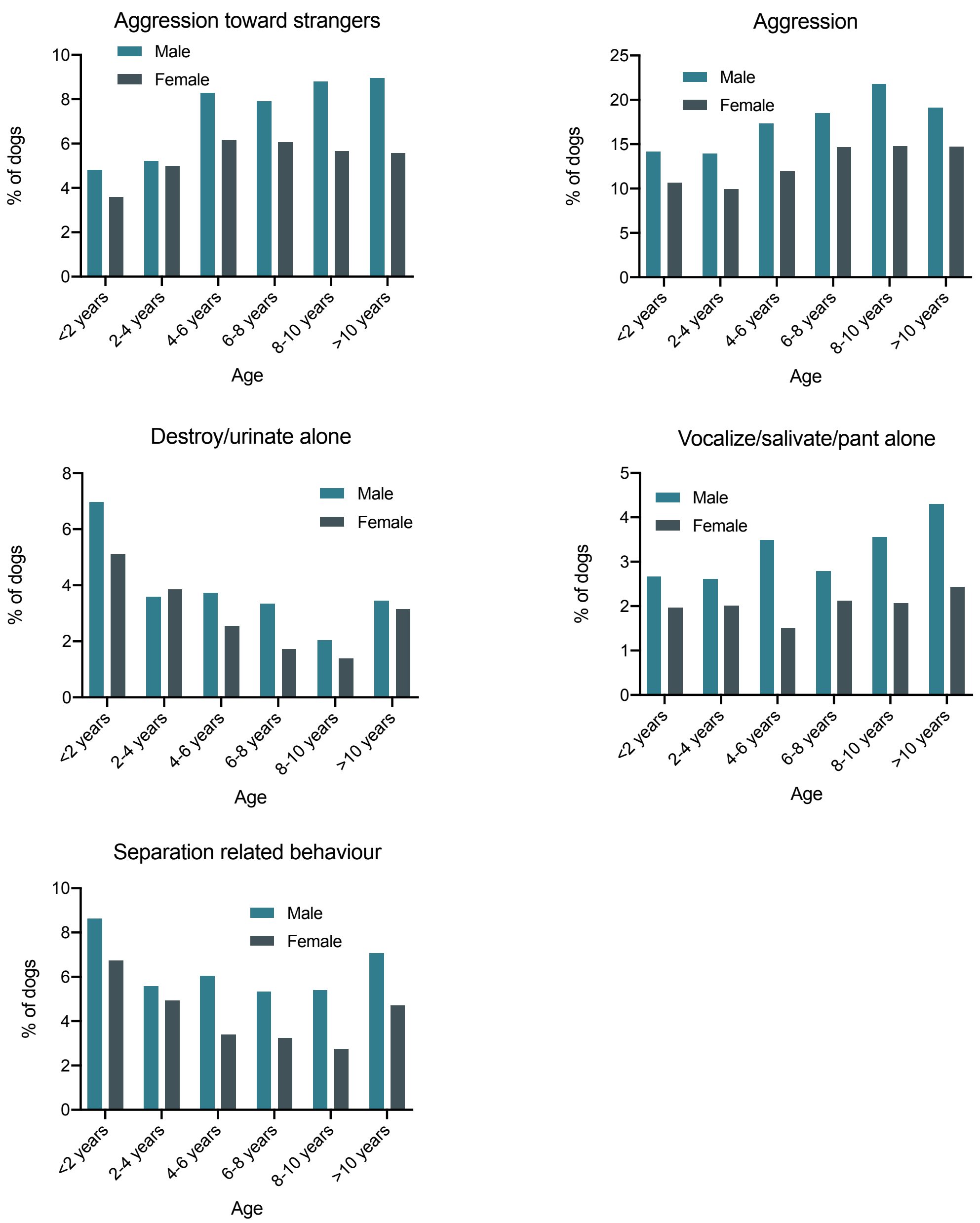
Aggression toward strangers
Male
Female
% of dogs
Age
<2 years
2-4 years
4-6 years
6-8 years
8-10 years
>10 years
Aggression
Destroy/urinate alone
Vocalize/salivate/pant alone
Separation related behaviour

Supplementary Figure S2. Breed differences of behaviour.

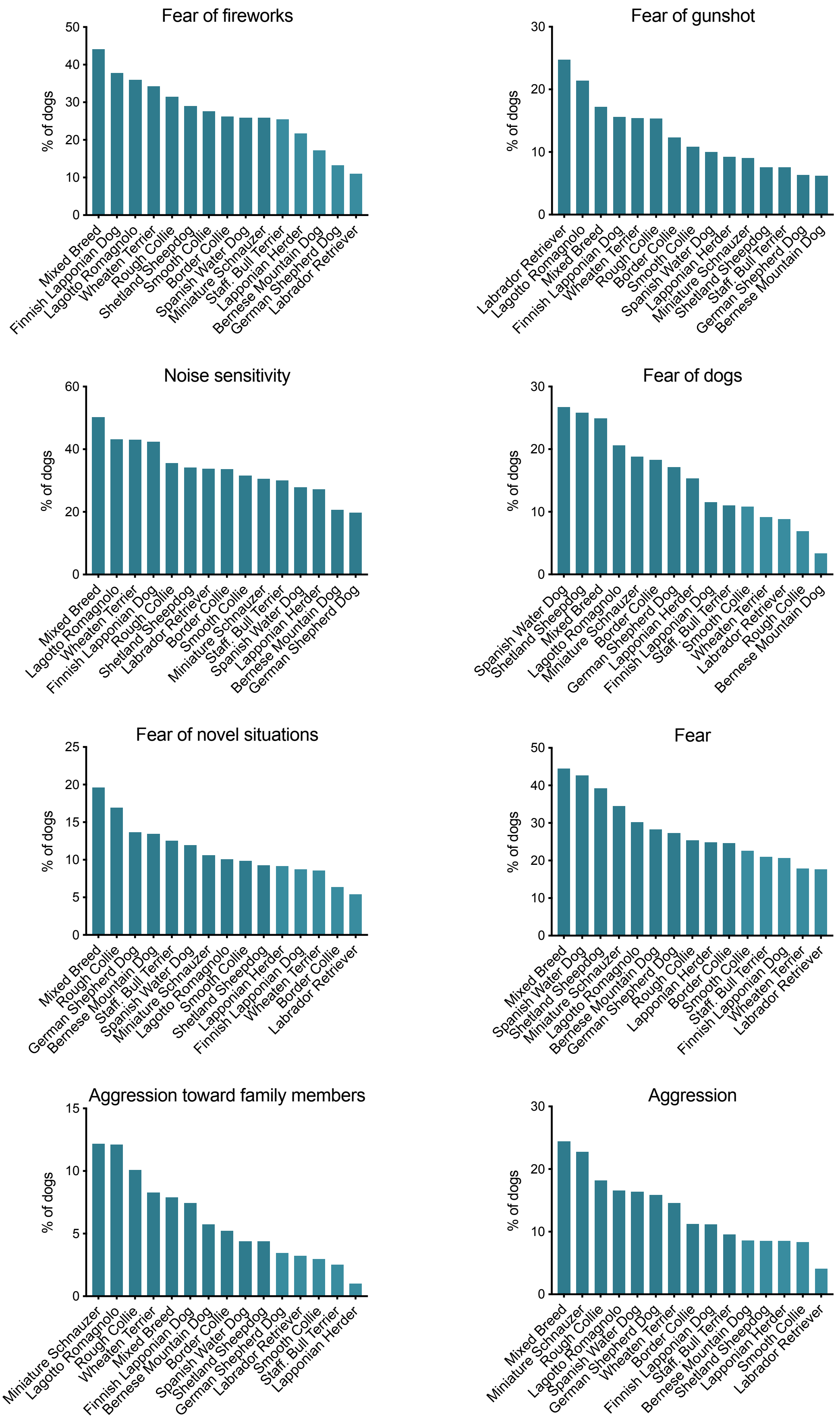

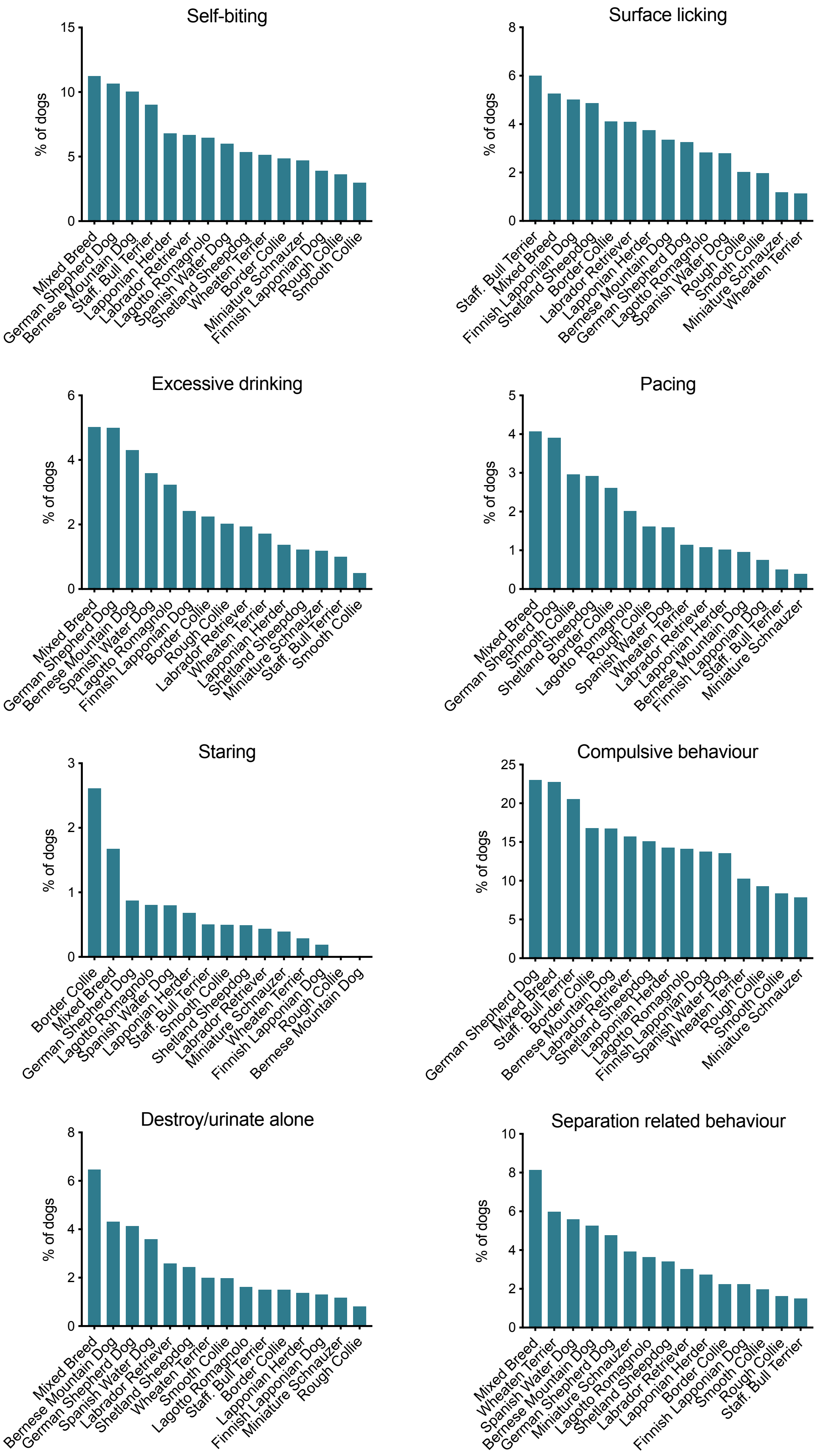

Self-biting
% of dogs
15
10
5
0
Mixed Breed
German Shepherd Dog
Bernese Mountain Dog
Staff. Bull Terrier
Lapponian Herder
Labrador Retriever
Lagotto Romagnolo
Spanish Water Dog
Shetland Sheepdog
Wheaten Terrier
Border Collie
Miniature Schnauzer
Finnish Lapponian Dog
Rough Collie
Smooth Collie
Surface licking
% of dogs
8
6
4
2
0
Staff. Bull Terrier
Mixed Breed
Finnish Lapponian Dog
Shetland Sheepdog
Border Collie
Labrador Retriever
Lapponian Herder
Bernese Mountain Dog
German Shepherd Dog
Lagotto Romagnolo
Spanish Water Dog
Rough Collie
Smooth Collie
Miniature Schnauzer
Wheaten Terrier
Excessive drinking
% of dogs
6
4
2
0
Mixed Breed
German Shepherd Dog
Bernese Mountain Dog
Spanish Water Dog
Lagotto Romagnolo
Finnish Lapponian Dog
Border Collie
Rough Collie
Labrador Retriever
Wheaten Terrier
Lapponian Herder
Shetland Sheepdog
Miniature Schnauzer
Staff. Bull Terrier
Smooth Collie
Pacing
% of dogs
5
4
3
2
1
0
Mixed Breed
German Shepherd Dog
Smooth Collie
Shetland Sheepdog
Border Collie
Lagotto Romagnolo
Rough Collie
Spanish Water Dog
Wheaten Terrier
Labrador Retriever
Lapponian Herder
Bernese Mountain Dog
Finnish Lapponian Dog
Staff. Bull Terrier
Miniature Schnauzer
Staring
% of dogs
3
2
1
0
Border Collie
Mixed Breed
German Shepherd Dog
Lagotto Romagnolo
Spanish Water Dog
Lapponian Herder
Staff. Bull Terrier
Smooth Collie
Shetland Sheepdog
Labrador Retriever
Miniature Schnauzer
Wheaten Terrier
Finnish Lapponian Dog
Rough Collie
Bernese Mountain Dog
Compulsive behaviour
% of dogs
25
20
15
10
5
0
German Shepherd Dog
Mixed Breed
Staff. Bull Terrier
Border Collie
Bernese Mountain Dog
Labrador Retriever
Shetland Sheepdog
Lapponian Herder
Lagotto Romagnolo
Finnish Lapponian Dog
Spanish Water Dog
Wheaten Terrier
Rough Collie
Smooth Collie
Miniature Schnauzer
Destroy/urinate alone
% of dogs
8
6
4
2
0
Mixed Breed
Bernese Mountain Dog
German Shepherd Dog
Spanish Water Dog
Labrador Retriever
Shetland Sheepdog
Wheaten Terrier
Smooth Collie
Lagotto Romagnolo
Staff. Bull Terrier
Border Collie
Lapponian Herder
Finnish Lapponian Dog
Miniature Schnauzer
Rough Collie
Separation related behaviour
% of dogs
10
8
6
4
2
0
Mixed Breed
Wheaten Terrier
Spanish Water Dog
Bernese Mountain Dog
German Shepherd Dog
Miniature Schnauzer
Lagotto Romagnolo
Shetland Sheepdog
Labrador Retriever
Lapponian Herder
Border Collie
Finnish Lapponian Dog
Smooth Collie
Rough Collie
Staff. Bull Terrier

Supplementary Figure S3. Radar chart representation of the behaviour of dog breeds. The minimum in all traits is the breed-wise minimum prevalence and the maximum in all traits is the breed-wise maximum prevalence. For the minimum and maximum prevalences, see Supplementary Table S7.

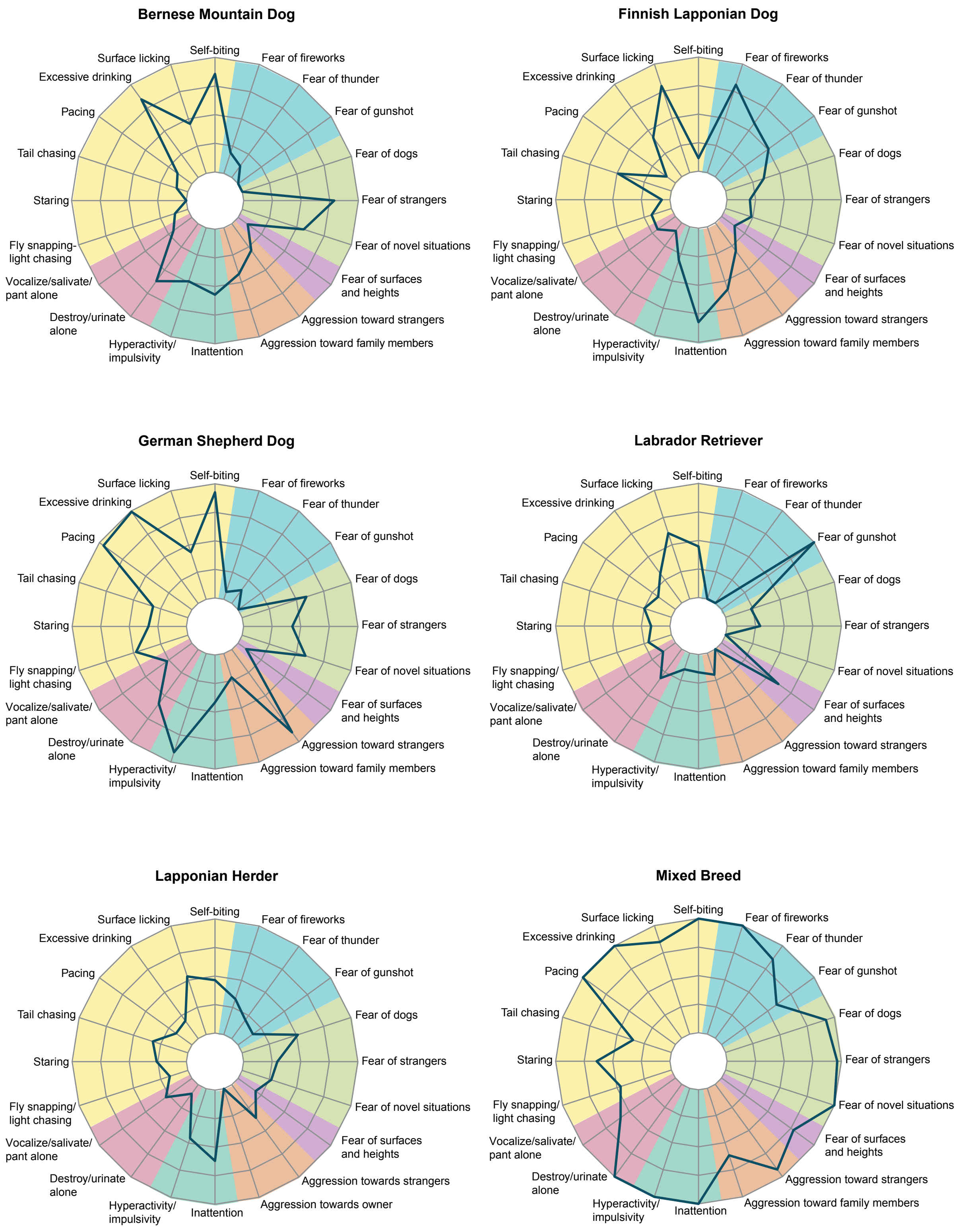

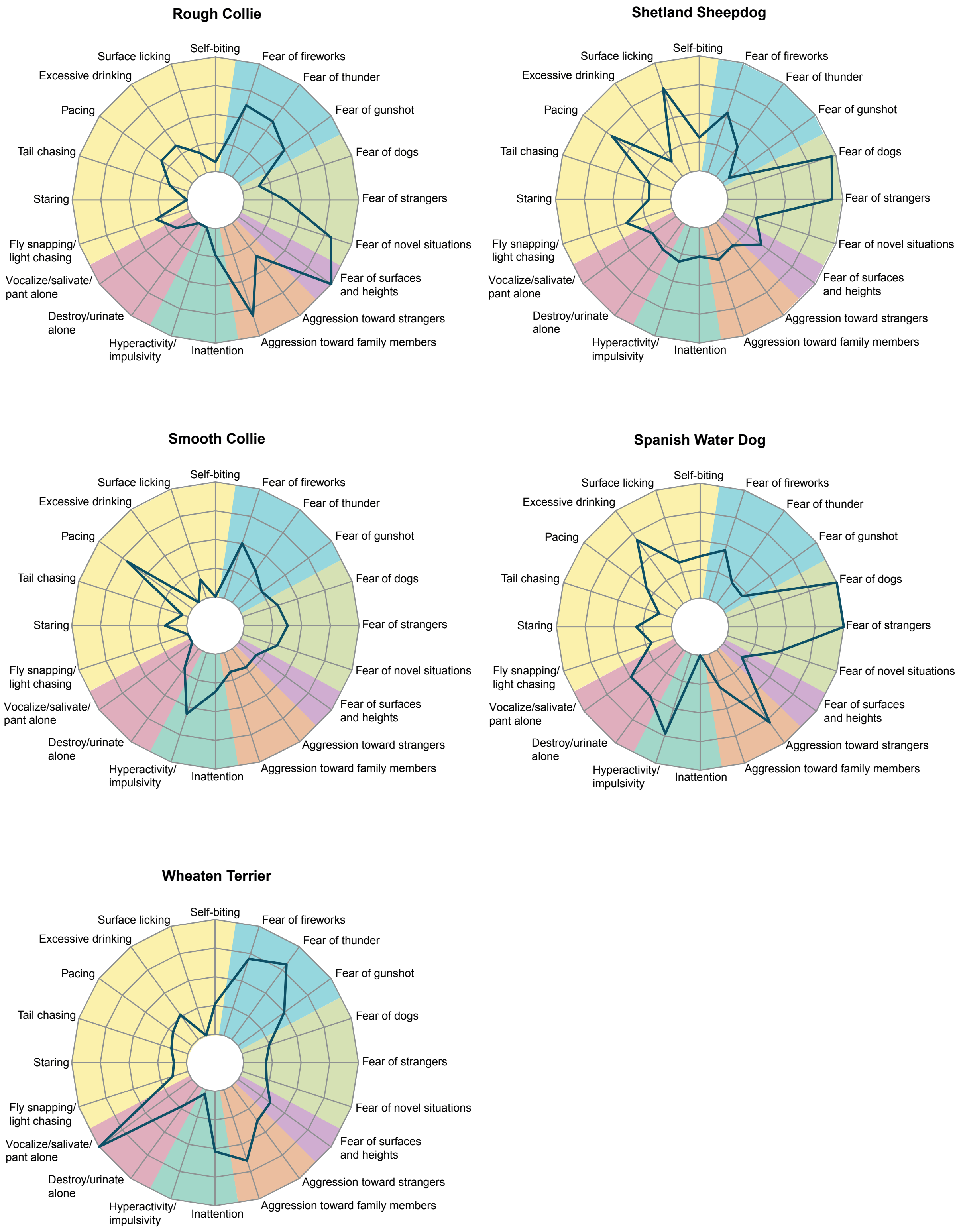
Rough Collie
Self-biting
Fear of fireworks
Fear of thunder
Fear of gunshot
Fear of dogs
Fear of strangers
Fear of novel situations
Fear of surfaces and heights
Aggression toward strangers
Aggression toward family members
Inattention
Hyperactivity/ impulsivity
Destroy/urinate alone
Vocalize/salivate/ pant alone
Fly snapping/ light chasing
Staring
Tail chasing
Pacing
Excessive drinking
Surface licking
Shetland Sheepdog
Self-biting
Fear of fireworks
Fear of thunder
Fear of gunshot
Fear of dogs
Fear of strangers
Fear of novel situations
Fear of surfaces and heights
Aggression toward strangers
Aggression toward family members
Inattention
Hyperactivity/ impulsivity
Destroy/urinate alone
Vocalize/salivate/ pant alone
Fly snapping/ light chasing
Staring
Tail chasing
Pacing
Excessive drinking
Surface licking
Smooth Collie
Self-biting
Fear of fireworks
Fear of thunder
Fear of gunshot
Fear of dogs
Fear of strangers
Fear of novel situations
Fear of surfaces and heights
Aggression toward strangers
Aggression toward family members
Inattention
Hyperactivity/ impulsivity
Destroy/urinate alone
Vocalize/salivate/ pant alone
Fly snapping/ light chasing
Staring
Tail chasing
Pacing
Excessive drinking
Surface licking
Spanish Water Dog
Self-biting
Fear of fireworks
Fear of thunder
Fear of gunshot
Fear of dogs
Fear of strangers
Fear of novel situations
Fear of surfaces and heights
Aggression toward strangers
Aggression toward family members
Inattention
Hyperactivity/ impulsivity
Destroy/urinate alone
Vocalize/salivate/ pant alone
Fly snapping/ light chasing
Staring
Tail chasing
Pacing
Excessive drinking
Surface licking
Wheaten Terrier
Self-biting
Fear of fireworks
Fear of thunder
Fear of gunshot
Fear of dogs
Fear of strangers
Fear of novel situations
Fear of surfaces and heights
Aggression toward strangers
Aggression toward family members
Inattention
Hyperactivity/ impulsivity
Destroy/urinate alone
Vocalize/salivate/ pant alone
Fly snapping/ light chasing
Staring
Tail chasing
Pacing
Excessive drinking
Surface licking

Supplementary information: online questionnaire

CANINE BEHAVIORAL QUESTIONNAIRE

We have developed a comprehensive behavioral questionnaire for dogs in all breeds and personality types. This questionnaire is designed to assess the temperament of dogs in many situations, including reaction to loud noises and strange people. Dogs of any age, breed, and temperament can participate in the study and therefore we encourage every dog owner to answer the questionnaire!

The purpose of this behavioral survey is to profile the behavioral patterns across breeds, to measure behavioral variability, and to identify dogs for genetic analyses to understand inherited and environmental factors that affect various behavior traits. The study can provide new information about the biological regulation of behavioral traits and may offer tools for better understanding and development of canine welfare.

Please fill out this online questionnaire carefully for each dog. The identity of you and your dog remains confidential. Answering takes about 20 minutes per dog.

This behavioral questionnaire is partly based on previously validated questionnaires (Tiira K & Lohi H, 2014; Vas et al. 2007, Wright et al. 2011).

Contact information

*First name of the owner:
*Surname of the owner:
Street address:
Postal code:
City:
*Phone number:
Email address:

*Breed of the dog:
*Dog's name (official):
Registration number:
*Birth year of the dog:
Birth month of the dog:
Birth date of the dog:
Time of death (yyyy.mm.dd):
*Sex of the dog

- o male
- o female

Has the dog been spayed/neutered

- o Yes
- o No

If you answered YES, at what age is your dog spayed/neutered?

Background information

Many behaviours are affected not only by genes but also by the dog's experiences, particularly in early life. Please take time to answer the following questions about your dog's history. You may have to contact your dog's breeder to be able to answer some of the questions.

*1. At what age did your dog enter your household?

2. Did the dog have any previous owners? Why didn't the previous owners keep the dog?

3. Separation from the mother or surrogate mother - at what age? This happens when the puppy is permanently separated from its mother of surrogate mother.
At what age was your dog separated from its mother or surrogate mother? Usually this happens at around 7-9 weeks, but it can also happen much earlier:

- under 4 weeks
- at the age of 4 weeks
- at the age of 5 weeks
- at the age of 6 weeks
- at the age of 7 weeks
- at the age of 8 weeks
- at the age of 9 weeks
- at the age of 10-12 weeks
- at over 12 weeks of age or older
- still living in the same household with its mother
- I don't know

How did the mother of your dog take care of the puppies?

- I don't know
- Mother took extremely good care of the puppies and spent a lot of time with them
- Mother took good care of the puppies
- Mother took relatively good care of the puppies, but sometimes it had to encouraged/told to go spend time with the puppies
- At the beginning the mother spent some time with the puppies, but later started to avoid being with puppies
- Mother did not want to spend time with the puppies, even in the beginning, but nursed enough so the puppies were not taken from their mother
- Mother did not take care of the puppies; the puppies were taken to a surrogate mother, or they were bottle-fed

How did the mother of your dog take care of the puppies? If none of the options is suitable, please describe the mother's behaviour.

4. The socialization period: has the dog experienced the following events during the period between 7 weeks and 4 months of age? How often? If you have obtained this dog later, move to the question number 5.
Met unfamiliar men

- very often (several times a day)
- often (twice a week-once a day)
- sometimes (twice a month-twice a week)
- seldom (1-2 times during puppyhood to 2 times per month)

- rarely (1-2 times during puppyhood or less)
- never

Met unfamiliar women

- very often (several times a day)
- often (twice a week-once a day)
- sometimes (twice a month-twice a week)
- seldom (1-2 times during puppyhood to 2 times per month)
- rarely (1-2 times during puppyhood or less)
- never

Met unfamiliar children

- very often (several times a day)
- often (twice a week-once a day)
- sometimes (twice a month-twice a week)
- seldom (1-2 times during puppyhood to 2 times per month)
- rarely (1-2 times during puppyhood or less)
- never

Met unfamiliar adult dogs

- very often (several times a day)
- often (twice a week-once a day)
- sometimes (twice a month-twice a week)
- seldom (1-2 times during puppyhood to 2 times per month)
- rarely (1-2 times during puppyhood or less)
- never

Visited city (or other place with traffic & many people)

- very often (several times a day)
- often (twice a week-once a day)
- sometimes (twice a month-twice a week)
- seldom (1-2 times during puppyhood to 2 times per month)
- rarely (1-2 times during puppyhood or less)
- never

Travelled by car

- very often (several times a day)
- often (twice a week-once a day)
- sometimes (twice a month-twice a week)
- seldom (1-2 times during puppyhood to 2 times per month)
- rarely (1-2 times during puppyhood or less)
- never

Travelled by bus

- very often (several times a day)
- often (twice a week-once a day)
- sometimes (twice a month-twice a week)
- seldom (1-2 times during puppyhood to 2 times per month)
- rarely (1-2 times during puppyhood or less)
- never

5. Your dog lives

- ○ Indoors
- ○ Outdoors (leashed/in a kennel)
- ○ Partially indoors, partially outdoors
- ○ Other

6 a. The number of adults in your family:
6 b. The number of children in your family:

*7. How many dogs you have not including this dog?
If you have more than one dog, please mention breed, sex, and age of the other dogs

*8. Is the dog in question your first? Second? 10th?

9. Exercise
How many times does your dog get exercise in a typical day?

- ○ three times or more
- ○ twice a day
- ○ once a day
- ○ dog is outside all the time
- ○ something else

If you answered 'something else', please specify
How many hours/minutes does your dog get exercise in a typical day?

- ○ three hours or more
- ○ 2-3 hours
- ○ 1-2 hours
- ○ 30 min-1 hour
- ○ less than 30 min

Exercise - on leash or running free?
During the daily walks, is your dog

- ○ on the leash during the whole walk
- ○ the dog is on the leash for part of the walk and allowed to run free for part of the walk
- ○ dog is mostly allowed to run free during the walks

10. Hobbies/sports/activities. Do you participate in some activities with your dog? What? (You can select multiple options.)

- ☐ Dog shows
- ☐ Obedience training
- ☐ Agility
- ☐ Tracking, protection etc.
- ☐ Search and rescue dog training
- ☐ Water rescue
- ☐ Hunting trials
- ☐ Hunting in practice
- ☐ Skijoring
- ☐ Herding
- ☐ Something else

How often do you do these activities with your dog (hobbies altogether)? In this we mean e.g. agilility or obedience, not daily exercise.

- never
- once a year
- 1-2 times during six months
- 1-2 times a month
- 1-2 times a week
- 2-4 times a week
- almost every day
- several times per day

11. Feeding

- What kind of food does your dog mainly eat?
- Raw food/BARF
- Partially raw food, partially dog food
- Homemade food - made for the dog
- Homemade food - table scraps
- Dog food purchased from pet store
- Dog food purchased from grocery store/supermarket
- Something else

If you answered something else, please specify what you feed to your dog.
Does your dog get any supplements?

- Regularly
- Sometimes
- Never

*12. Being alone
How much time does your dog spend alone in the house/kennel during a typical working day?

- 0 hours
- 0-1 hours
- 1-3 hours
- 3-6 hours
- 6-8 hours
- 8-9 hours
- 9-10 hours
- 10 hours or more
- I don't know

Shyness/fearfulness

The following questions deal with your dog’s potential fearful reactions towards strangers, unfamiliar dogs, and new/strange places or situations.

If your dog’s behaviour has changed at some point, please describe that change in the section ‘Changed behaviour’.

STRANGER. Think how your dog usually reacts when meeting a stranger. If your dog shows shyness towards strangers, please mark YES, my dog shows fearful behaviour, and then mark those behaviour(s) that best describe the reaction of your dog. If none of the behaviours listed below describe well your dog's reaction, you can add a suitable behaviour to the list. If your dog does not behave fearfully, mark NO, and mark how your dog does behave when meeting a stranger.

13. STRANGER

*Does your dog show shyness or fear when meeting a strange person

- ○ YES
- ○ NO
- ○ I don't know

If you answered YES, please mark one or more behaviours that describe your dog's reaction

- ☐ withdraws
- ☐ barks (does not go towards the person)
- ☐ growls (does not go towards the person)
- ☐ tail low / between the legs
- ☐ not willing to make contact
- ☐ stays close to the owner (even when not under a command)
- ☐ barks / growls AND goes towards a stranger
- ☐ something else

  If you answered 'something else', please specify

If you answered yes, how often does the dog react fearfully?

My dog shows shyness or fearful behaviour towards stanger

- ○ Always, 100% of the times
- ○ Almost always, 60-100% of the times
- ○ Often, 40-60% of the times
- ○ Sometimes, 20-40% of the times
- ○ Rarely, 0-20% of the times

If you answered NO, please specify how your dog behaves when meeting a stranger

- ☐ if allowed, always goes to greet the person
- ☐ jumps, licks, is very excited
- ☐ sniffs, tail is wagging, and is relaxed
- ☐ makes contact, but loses interest soon
- ☐ is not interested in people, but does not withdraw or mind if a person wants to pet it
- ☐ is not interested, is quite indifferent
- ☐ barks, growls
- ☐ something else

  If you answered 'something else', please specify

Changed behaviour - former behaviour:

Changed behaviour - present behaviour:

UNFAMILIAR DOG. Think how your dog usually reacts when meeting unfamiliar dogs. If your dog is shy when meeting unfamiliar dogs, please mark YES, my dog shows fearful reaction, and then mark those appropriate behaviour(s) which best describe the reaction of your dog. If none of the behaviours listed below describe well your dog's reaction, you can add a suitable behaviour to the list. If your dog does not behave fearfully, mark NO, and mark how your dog does behave.

14. UNFAMILIAR DOG
*Does your dog show shyness or fear when meeting an unfamiliar dog?
- ○ YES
- ○ NO
- ○ I don't know

If you answered YES, please mark one or more behaviours that describe your dog's reaction
- ☐ withdraws
- ☐ barks (does not go towards the dog)
- ☐ growls (does not go towards the dog)
- ☐ tail low / between the legs
- ☐ not willing to make contact
- ☐ stays close to the owner (even when not under a command)
- ☐ barks / growls AND goes towards a strange dog
- ☐ something else

  If you answered 'something else', please specify

If you answered yes, how often does the dog react fearfully?
My dog shows shyness or fearful behaviour towards a strange dog
- ○ Always, 100% of the times
- ○ Almost always, 60-100% of the times
- ○ Often, 40-60% of the times
- ○ Sometimes, 20-40% of the times
- ○ Rarely, 0-20% of the times

If you answered NO, please specify how your dog behaves when meeting an unfamiliar dog
- ☐ eager to meet, always friendly
- ☐ eager to meet, but loses its interest soon
- ☐ usually friendly, but responds aggressively if another dog shows aggressive behaviour
- ☐ usually friendly, but sometimes starts a fight
- ☐ usually growls
- ☐ usually barks
- ☐ barks / growls AND goes towards a dog
- ☐ indifferent, not interested in other dogs
- ☐ something else

  If you answered 'something else', please specify

Changed behaviour - former behaviour:
Changed behaviour - present behaviour:

NEW SITUATION, NEW ENVIRONMENT. Consider how your dog usually reacts to new places or situations. If your dog shows fear or is stressed (panting, trembling, abnormally flaky skin) please mark YES, and then mark those appropriate behaviour(s) which best describe the behaviour of your dog. If none of the behaviours listed below describe well your dog's behaviour, you can add a suitable behaviour to the list. Then mark how your dog does behave.

If the dog is fearful only at the vet, please ignore it and answer how your dog reacts to other situations and environments.

15. NEW SITUATION, NEW ENVIRONMENT
*Does your dog show fear or stress in a new situation or in a new environment?
- ○ YES
- ○ NO
- ○ I don't know

If you answered YES, please mark one or more behaviours that describe your dog's reaction
wants out of the situation / new environment

- ☐ barks
- ☐ tail low or between the legs
- ☐ stays still, does not want to explore the new environment
- ☐ stays close the owner (even when not under a command)
- ☐ walks low
- ☐ pants
- ☐ trembles
- ☐ something else

  If you answered 'something else', please specify

How often does the dog react fearfully?

- ○ Always, 100% of the times
- ○ Almost always, 60-100% of the times
- ○ Often, 40-60% of the times
- ○ Sometimes, 20-40% of the times
- ○ Rarely, 0-20% of the times

If you answered NO, please specify how your dog behaves in a new situation or environment

- ☐ tail in high position
- ☐ tail below the backline, but not between the legs
- ☐ dog is curious, eager to inspect
- ☐ is able to eat and sleep in the new place
- ☐ is calm, rather indifferent
- ☐ something else

  If you answered 'something else', please specify

Changed behaviour - former behaviour:
Changed behaviour - present behaviour:

Other things that might be related to your dog’s behaviour towards strange persons or dogs, or to its behaviour in new situations:

Aggressiveness

The following questions deal with your dog’s aggressive behaviour towards strangers, the owner/family members, and other dogs. Please mark the frequency of the behaviour.

16. STRANGER
*The dog barks when the doorbell rings or the door is knocked

- ○ Never
- ○ Rarely
- ○ Sometimes
- ○ Often
- ○ Always or almost always
- ○ I don't know

*The dog barks when strange people come in and at first does not want to greet

- ○ Never
- ○ Rarely
- ○ Sometimes

- Often
- Always or almost always
- I don't know

*The dog barks when a stranger tries to touch or pet it in its home

- Never
- Rarely
- Sometimes
- Often
- Always or almost always
- I don't know

*The dog growls when a stranger tries to touch or pet it in its home

- Never
- Rarely
- Sometimes
- Often
- Always or almost always
- I don't know

*The dog tries to snap or bite when a stranger tries to touch or pet it in its home

- Never
- Rarely
- Sometimes
- Often
- Always or almost always
- I don't know

*The dog is happy and excited when strangers come to its home

- Never
- Rarely
- Sometimes
- Often
- Always or almost always
- I don't know

*When a stranger wants to pet the dog outside the home and the dog is on a leash, my dog barks at the stranger

- Never
- Rarely
- Sometimes
- Often
- Always or almost always
- I don't know

*When a stranger wants to pet the dog outside the home and the dog is on a leash, my dog growls at the stranger

- Never
- Rarely
- Sometimes
- Often

- Always or almost always
- I don't know

*When a stranger wants to pet the dog outside the home and the dog is on a leash, my dog tries to snap or bite the stranger

- Never
- Rarely
- Sometimes
- Often
- Always or almost always
- I don't know

*The dog barks or growls in the car at passersby

- Never
- Rarely
- Sometimes
- Often
- Always or almost always
- I don't know

17. OWNER/FAMILY MEMBER

*When the owner/family member handles (washes, grooms, cuts the nails etc.) the dog, it growls

- Never
- Rarely
- Sometimes
- Often
- Always or almost always
- I don't know

*When the owner/family member handles (washes, grooms, cuts the nails etc.) the dog, it tries to snap or bite

- Never
- Rarely
- Sometimes
- Often
- Always or almost always
- I don't know

*When the owner/family member takes a bone/food/toy from the dog, it growls

- Never
- Rarely
- Sometimes
- Often
- Always or almost always
- I don't know

*When the owner/family member takes a bone/food/toy from the dog, it tries to snap or bite

- Never
- Rarely
- Sometimes

- Often
- Always or almost always
- I don't know

Are there other situations, when your dog behaves aggressively? Please specify:

Changed behaviour - former behaviour
Changed behaviour - present behaviour

Noise phobia

The following questions deal with your dog's reaction to loud noises. If your dog does not react to a particular noise, mark NO. If your dog's behaviour has changed at any time, please describe the former and present behaviour under 'Changed behaviour'.

19. How often does the dog hear the following sounds?
Thunder
- Never
- A few times a year
- About once a month
- Several times a month

Fireworks
- Never
- A few times a year
- About once a month
- Several times a month

Gunfire
- Never
- A few times a year
- About once a month
- Several times a month

THUNDERSTORM
*20. Does your dog react to a thunderstorm?
- YES
- NO
- I don't know

If you answered YES, please mark one or more behaviours that desribe your dog's reaction. If you answered NO, move to the question 21.
- [ ] salivates
- [ ] defecates
- [ ] urinates
- [ ] destroys
- [ ] escapes
- [ ] pants
- [ ] hides
- [ ] trembles

- ☐ vocalizes
- ☐ paces
- ☐ freezes
- ☐ tail low / between legs
- ☐ the dog gets excited when hearing thunder, the tail is up, may bark
- ☐ the dog gets irritable when hearing thunder, it can bark and go towards the noise (does not seem timid)

How often does your dog react as indicated above?
My dog reacts...

- ○ Always, 100% of the times
- ○ Almost always, 60-100% of the times
- ○ Often, 40-60% of the times
- ○ Sometimes 20-40% of the times
- ○ Rarely, 0-20% of the times

Changed behaviour - former behaviour:
Changed behaviour - present behaviour:
At what age did the dog's possible fearful reaction towards loud noises started?

FIREWORKS
*21. Does your dog react to FIREWORKS?

- ○ YES
- ○ NO
- ○ I don't know

If you answered YES, please mark one or more behaviours that describe your dog's reaction. If you answered 'NO', jump to the question 22.

- ☐ salivates
- ☐ defecates
- ☐ urinates
- ☐ destroys
- ☐ escapes
- ☐ pants
- ☐ hides
- ☐ trembles
- ☐ vocalizes
- ☐ paces
- ☐ freezes
- ☐ tail low / between legs
- ☐ the dog gets excited when hearing fireworks, barks and/or tries to chase them
- ☐ the dog gets irritable when hearing fireworks, it can bark and go towards the noise (does not seem timid)

How often does your dog react as indicated above?
My dog reacts...

- ○ Always, 100% of the times
- ○ Almost always, 60-100% of the times
- ○ Often, 40-60% of the times
- ○ Sometimes, 20-40% of the times
- ○ Rarely, 0-20% of the times

Changed behaviour - former behaviour:
Changed behaviour - present behaviour:

At what age did the dog's possible fearful reaction towards loud noises started?

GUNFIRE
*22. Does your dog react to gunfire?
- ○ YES
- ○ NO
- ○ I don't know

If you answered YES, please mark one or more behaviours that desribe your dog's reaction. If you answered 'NO', jump to the question 23.
- ☐ salivates
- ☐ defecates
- ☐ urinates
- ☐ destroys
- ☐ escapes
- ☐ pants
- ☐ hides
- ☐ trembles
- ☐ vocalizes
- ☐ paces
- ☐ freezes
- ☐ tail low / between legs
- ☐ the dog gets excited when hearing gunfire.
- ☐ the dog gets irritable when hearing gunfire, it can bark and go towards the noise (does not seem timid)

How often does your dog react as indicated above?
My dog reacts...
- ○ Always, 100% of the times
- ○ Almost always, 60-100% of the times
- ○ Often, 40-60% of the times
- ○ Sometimes, 20-40% of the times
- ○ Rarely, 0-20% of the times

Changed behaviour - former behaviour:
Changed behaviour - present behaviour:
At what age did the dog's possible fearful reaction towards loud noises started?

OTHER NOISES
*23. Does your dog react to other noises (vacuum cleaners, leaf blowers, sirens, alarm systems, etc.)?
- ○ YES
- ○ NO
- ○ I don't know

If you answered YES, please mark one or more behaviours that desribe your dog's reaction
- ☐ salivates
- ☐ defecates
- ☐ urinates
- ☐ destroys
- ☐ escapes
- ☐ pants
- ☐ hides
- ☐ trembles

- ☐ vocalizes
- ☐ paces
- ☐ freezes
- ☐ tail low / between legs
- ☐ the dog gets excited when hearing other noises.
- ☐ the dog gets irritable when hearing other noises, it can bark and go towards the noise (does not seem timid).

How often does your dog react as indicated above?
My dog reacts...

- ○ Always, 100% of the times
- ○ Almost always, 60-100% of the times
- ○ Often, 40-60% of the times
- ○ Sometimes, 20-40% of the times
- ○ Rarely, 0-20% of the times

Changed behaviour - former behaviour:
Changed behaviour - present behaviour:

Answer the following questions ONLY if your dog is afraid of loud noises
How long does the dog's fearful reaction last after the thunder has stopped? The dog returns to normal

- ○ right after the sound has stopped
- ○ in a few minutes after the sound has stopped
- ○ in 15 mins-1 hour after the sound has stopped
- ○ in 1-5 hours after the sound has stopped
- ○ in more than 5 hours
- ○ I don't know

How long does the dog's fearful reaction last after the fireworks have stopped? The dog returns to normal

- ○ right after the sound has stopped
- ○ in a few minutes after the sound has stopped
- ○ in 15 mins-1 hour after the sound has stopped
- ○ in 1-5 hours after the sound has stopped
- ○ in more than 5 hours
- ○ I don't know

How long does the dog's fearful reaction last after the gunfire has stopped? The dog returns to normal

- ○ right after the sound has stopped
- ○ in a few minutes after the sound has stopped
- ○ in 15 mins-1 hour after the sound has stopped
- ○ in 1-5 hours after the sound has stopped
- ○ in more than 5 hours
- ○ I don't know

How long does the dog's fearful rection last after other noise (vacuum cleaner, leaf blower, siren, alarm system, etc.) has stopped? The dog returns to normal

- ○ right after the sound has stopped
- ○ in a few minutes after the sound has stopped
- ○ in 15 mins-1 hour after the sound has stopped

- ○ in 1-5 hours after the sound has stopped
- ○ in more than 5 hours
- ○ I don't know

24. Has this dog ever been treated for noise sensitivities or phobias? If so, with what?
- ☐ Medications
- ☐ Natural products
- ☐ Desensitization, e.g. playing recordings of the sound that causes fear
- ☐ Something else
  If you answered 'something else', please specify

25. Do you have additional comments about your dog’s reaction to noises, or is there anything else about its behaviour when exposed to noise that you think could be relevant to our study?

Surfaces and high places

Following questions focus on your dogs behaviour when walking in various surfaces and in high places.

25. Surfaces and high places
Does your dog have difficulties to walk on a metal grid?
- ○ Yes, always
- ○ Often
- ○ Sometimes, depends on the place
- ○ Rarely
- ○ Never
- ○ I don't know

Does your dog have difficulties to walk on shiny floors (like in shopping malls, at veterinary clinics etc)?
- ○ Yes, always
- ○ Often
- ○ Sometimes, depends on the place
- ○ Rarely
- ○ Never
- ○ I don't know

Does your dog have difficulties to climb stairs where you can see ‘between’ the steps?
- ○ Yes, always
- ○ Often
- ○ Sometimes, depends on the place
- ○ Rarely
- ○ Never
- ○ I don't know

Does your dog have difficulties to climb stairs where you cannot see ‘between’ the steps?
- ○ Yes, always
- ○ Often
- ○ Sometimes, depends on the place

- Rarely
- Never
- I don't know

Does your dog have difficulties to walk next to glass railings, for example on the second floor in a shopping mall?

- Yes, always
- Often
- Sometimes, depends on the place
- Rarely
- Never
- I don't know

Does your dog have difficulties to climb metal stairs where you can see ‘through’ the steps?

- Yes, always
- Often
- Sometimes, depends on the place
- Rarely
- Never
- I don't know

Does your dog have difficulties to walk over narrow bridges?

- Yes, always
- Often
- Sometimes, depends on the place
- Rarely
- Never
- I don't know

Does your dog have difficulties to walk from one surface to another (for example from outside to inside in a new place)?

- Yes, always
- Often
- Sometimes, depends on the place
- Rarely
- Never
- I don't know

Does your dog have problems on other surfaces or in high places? If your dog has other problems with surfaces or high places which were not included in the questionnaire, please describe them below in your own words.

Separation anxiety

Separation anxiety
*Does your dog exhibit separation anxiety when left alone or left with other dogs?

- YES
- NO
- I don't know

How does your dog react when the owner is out of the house and the dog is home alone or with other dogs?

The dog destroys / chews on things when it is home alone

- ○ Never
- ○ Rarely
- ○ Sometimes
- ○ Often
- ○ Very often
- ○ I don't know

The dog urinates or defecates when it is home alone

- ○ Never
- ○ Rarely
- ○ Sometimes
- ○ Often
- ○ Very often
- ○ I don't know

The dog vocalizes (e.g. howls, barks) when it is home alone

- ○ Never
- ○ Rarely
- ○ Sometimes
- ○ Often
- ○ Very often
- ○ I don't know

The dog salivates when it is home alone

- ○ Never
- ○ Rarely
- ○ Sometimes
- ○ Often
- ○ Very often
- ○ I don't know

The dog pants when it is home alone

- ○ Never
- ○ Rarely
- ○ Sometimes
- ○ Often
- ○ Very often
- ○ I don't know

How do you know about your dog's reactions when it is home alone?

- ☐ I have videotaped the dog
- ☐ My neighbours have told me
- ☐ I read the dog's mood when I come home
- ☐ The dog has destroyed when being alone
- ☐ Something else

  If you answered 'something else', please specify

When did separation anxiety begin?

- ○ before 3 months of age
- ○ at 3 months - 6 months of age
- ○ at 6 months - 1 year of age
- ○ at 1 year - 1.5 year of age
- ○ at 1.5 - 2 years of age
- ○ at 2-3 years of age
- ○ older
  If the behaviour started when older, please specify the age

Hyperactivity/Impulsivity

Please answer how OFTEN the statement is true for your dog.
*My dog is hyperactive

- ○ I don't know
- ○ Never
- ○ Seldom
- ○ Sometimes
- ○ Often
- ○ Always or almost always

*My dog's hyperactivity/impulsiveness disturbs daily routines and/or training the dog

- ○ I don't know
- ○ Never
- ○ Seldom
- ○ Sometimes
- ○ Often
- ○ Always or almost always

When did hyperactive or impulsive behaviour begin?

- ○ before 3 months of age
- ○ at 3 months - 6 months of age
- ○ at 6 months - 1 year of age
- ○ at 1 year - 1.5 year of age
- ○ at 1.5 - 2 years of age
- ○ at 2-3 years of age
- ○ older
  If the behaviour started when older, please specify the age

Please note how often the statement is true for your dog.
1. My dog has a difficult time learning, because it is careless or other things can easily attract its attention.

- ○ Never
- ○ Sometimes
- ○ Often
- ○ Very often

2. It's easy to attract its attention, but it loses its interest soon.

- Never
- Sometimes
- Often
- Very often

3. It's difficult for it to concentrate on a task or play.

- Never
- Sometimes
- Often
- Very often

4. It leaves from its place when it should stay.

- Never
- Sometimes
- Often
- Very often

5. It cannot be quiet, it cannot be easily calmed.

- Never
- Sometimes
- Often
- Very often

6. It fidgets all the time.

- Never
- Sometimes
- Often
- Very often

7. It seems that it doesn't listen even if it knows that someone is speaking to it.

- Never
- Sometimes
- Often
- Very often

8. It is excessive, difficult to control, and if it lunges it is hard to hold back.

- Never
- Sometimes
- Often
- Very often

9. It would always play and run.

- Never
- Sometimes
- Often
- Very often

10. It solves simple tasks easily, but it often has difficulties with complicated tasks, even if it knows them and has practiced them often.

- Never
- Sometimes
- Often

- Very often

11. It is likely to react hastily and that's why it's failing tasks.
    - Never
    - Sometimes
    - Often
    - Very often
12. Its attention can be easily distracted.
    - Never
    - Sometimes
    - Often
    - Very often
13. It cannot wait as it has no self-control.
    - Never
    - Sometimes
    - Often
    - Very often

If you want, you may add short notes regarding the above statements. Please indicate the number of the statement in front of the comment.

Please indicate how well does the sentence describe your dog

1. My dog shows extreme physical signs when excited (e.g. drooling, panting, raising hackels, urination, licking lips, widening of eyes)
    - Strongly disagree
    - Generally disagree
    - Partly agree, partly disagree
    - Generally agree
    - Strongly agree
2. When my dog gets very excited it can lead to fixed repetitive behaviour (i.e., an action that is repeated in the same way over and over again), such as tail chasing or spinning around in circles
    - Strongly disagree
    - Generally disagree
    - Partly agree, partly disagree
    - Generally agree
    - Strongly agree
3. I would consider my dog to be very impulsive (i.e., has sudden, strong urges to act; acts without forethought; acts without considering effects of actions)
    - Strongly disagree
    - Generally disagree
    - Partly agree, partly disagree
    - Generally agree
    - Strongly agree
4. My dog doesn't like to be approached or hugged
    - Strongly disagree
    - Generally disagree
    - Partly agree, partly disagree
    - Generally agree

- Strongly agree

5. My dog becomes aggressive (e.g., growl, snarl, snap, bite) when excited
    - Strongly disagree
    - Generally disagree
    - Partly agree, partly disagree
    - Generally agree
    - Strongly agree
6. My dog appears to be 'sorry' after it has done something wrong
    - Strongly disagree
    - Generally disagree
    - Partly agree, partly disagree
    - Generally agree
    - Strongly agree
7. My dog does not think before it acts (e.g., would steal food without first looking to see if someone is watching)
    - Strongly disagree
    - Generally disagree
    - Partly agree, partly disagree
    - Generally agree
    - Strongly agree
8. My dog can be very persistent (e.g., will continue to do something even if it knows it will get punished or told off)
    - Strongly disagree
    - Generally disagree
    - Partly agree, partly disagree
    - Generally agree
    - Strongly agree
9. My dog may become aggressive (e.g., growl, snarl, snap, bite) if frustrated with something
    - Strongly disagree
    - Generally disagree
    - Partly agree, partly disagree
    - Generally agree
    - Strongly agree
10. My dog is easy to train
    - Strongly disagree
    - Generally disagree
    - Partly agree, partly disagree
    - Generally agree
    - Strongly agree
11. My dog is not keen to go into new situations
    - Strongly disagree
    - Generally disagree
    - Partly agree, partly disagree
    - Generally agree
    - Strongly agree

12. My dog takes a long time to lose interest in new things
    - Strongly disagree
    - Generally disagree
    - Partly agree, partly disagree
    - Generally agree
    - Strongly agree
13. My dog calms down very quickly after being excited
    - Strongly disagree
    - Generally disagree
    - Partly agree, partly disagree
    - Generally agree
    - Strongly agree
14. My dog appears to have a lot of control over how it responds
    - Strongly disagree
    - Generally disagree
    - Partly agree, partly disagree
    - Generally agree
    - Strongly agree
15. My dog is very interested in new things and new places
    - Strongly disagree
    - Generally disagree
    - Partly agree, partly disagree
    - Generally agree
    - Strongly agree
16. My dog reacts very quickly
    - Strongly disagree
    - Generally disagree
    - Partly agree, partly disagree
    - Generally agree
    - Strongly agree
17. My dog is not very patient (e.g., gets agitated waiting for its food, or waiting to go out for a walk)
    - Strongly disagree
    - Generally disagree
    - Partly agree, partly disagree
    - Generally agree
    - Strongly agree
18. My dog seems to get excited for no reason
    - Strongly disagree
    - Generally disagree
    - Partly agree, partly disagree
    - Generally agree
    - Strongly agree

If you want, you may add short notes regarding the above statements. Please indicate the number of the statement in front of the comment.

Stereotypic behaviour

Tail-chasing/spinning - The dog tries to catch its tail and spins. The dog may stop and stare at its tail or it may just spin fast or slow.
Does your dog chase his/her tail?

- I've never noticed this behaviour
- a few times during the dog's lifetime
- every once in a while (monthly-yearly)
- quite often (weekly-monthly)
- repeatedly (every other day-weekly)
- daily
- several times per day

Reflections, shadows and invisible things - The dog stares/chases reflections or shadows, or the dog looks like it is trying to catch invisible things.
Does your dog pursue, snatch, or bite at reflections, shadows or invisible things?

- I've never noticed this behaviour
- a few times during the dog's lifetime
- every once in a while (monthly-yearly)
- quite often (weekly-monthly)
- repeatedly (every other day-weekly)
- daily
- several times per day

What of the behaviours described (snapping reflections, shadows, or invisible things) does your dog express?

Licking - Dog may lick e.g. floors and walls
Does your dog lick different surfaces?

- I've never noticed this behaviour
- a few times during the dog's lifetime
- every once in a while (monthly-yearly)
- quite often (weekly-monthly)
- repeatedly (every other day-weekly)
- daily
- several times per day

Pattern/schematic (ex. circle, figure 8's) or "aimless" running/movement
Does your dog run/pace indoors/outdoors (e.g. in a kennel) in a fixated manner?

- I've never noticed this behaviour
- a few times during the dog's lifetime
- every once in a while (monthly-yearly)
- quite often (weekly-monthly)
- repeatedly (every other day-weekly)
- daily
- several times per day

Visual fixation behaviour - Dog may stare at one point for very long period, even if there is really nothing to see. A dog may stare at wall, ceiling etc.
Does your dog stare (into space) at a specific spot for long periods?

- I've never noticed this behaviour
- a few times during the dog's lifetime
- every once in a while (monthly-yearly)
- quite often (weekly-monthly)
- repeatedly (every other day-weekly)
- daily
- several times per day

Drinking too much or repeatedly visiting the water bowl (you can answer even if you feel that your dog's drinking behaviour is normal)
Have you had to prevent your dog from going to the water bowl?

- Yes
- No

Drinking too much or repeatedly visiting the water bowl: How much time spent?
How much time does your dog spend daily on behaviour related to the water bowl (drinking, standing near it etc)?

- less than 5 min
- 5-15 min
- 15min - 30 min
- 30 min - 1 hour
- 1 hour or more

Describe a typical episode where your dog repeatedly goes to the water bowl or drinks too much:

Does your dog bite themselves (paws, flank or other body part)?

- Never
- Sometimes
- Almost every day
- Several hours per day

Does your dog have some other "weird" or "abnormal" behaviours? Describe below:

Does your dog have noticeable wounds due to the behaviour (such as on the tail from chasing/biting, skin wounds from licking, or dental wounds from e.g. stone carrying)?

- Yes
- No
- I don't know

  If you answered yes, please describe

Is your dog given medication for stereotypic behaviour?

- Yes
- No

  If you answered yes, please write down the name of the medication
